# Mesenchymal stem cells, as glioma exosomal immunosuppressive signal multipliers, enhance MDSCs immunosuppressive activity through the miR-21/SP1/DNMT1 positive feedback loop

**DOI:** 10.1186/s12951-023-01997-x

**Published:** 2023-07-22

**Authors:** Wei Qiu, Qindong Guo, Xiaofan Guo, Chaochao Wang, Boyan Li, Yanhua Qi, Shaobo Wang, Rongrong Zhao, Xiao Han, Hao Du, Shulin Zhao, Ziwen Pan, Yang Fan, Qingtong Wang, Zijie Gao, Gang Li, Hao Xue

**Affiliations:** 1 Department of Neurosurgery, Qilu Hospital, Cheeloo College of Medicine and Institute of Brain and Brain-Inspired Science, Shandong University, 107 Wenhua Western Road, Jinan, Shandong 250012 China; 2 Shandong Key Laboratory of Brain Function Remodeling, Jinan, Shandong China; 3 Department of Neurology, Loma Linda University Health, Loma Linda, CA 92350 USA; 4 Department of Neurosurgery, Qilu Hospital of Shandong University (Qingdao), Qingdao, Shandong China; 5 Department of Neurosurgery, Jinan Children’s Hospital, Jinan, Shandong China; 6 Department of Cell Biology, University of Connecticut School of Medicine, Farmington, CT 06030 USA

**Keywords:** Glioma, Mesenchymal stem cells, Myeloid-derived suppressor cells, Exosomes, miR-21, Anti-PD-1 therapy

## Abstract

**Background:**

The immunosuppressive microenvironment in glioma induces immunotherapy resistance and is associated with poor prognosis. Glioma-associated mesenchymal stem cells (GA-MSCs) play an important role in the formation of the immunosuppressive microenvironment, but the mechanism is still not clear.

**Results:**

We found that GA-MSCs promoted the expression of CD73, an ectonucleotidase that drives immunosuppressive microenvironment maintenance by generating adenosine, on myeloid-derived suppressor cells (MDSCs) through immunosuppressive exosomal miR-21 signaling. This process was similar to the immunosuppressive signaling mediated by glioma exosomal miR-21 but more intense. Further study showed that the miR-21/SP1/DNMT1 positive feedback loop in MSCs triggered by glioma exosomal CD44 upregulated MSC exosomal miR-21 expression, amplifying the glioma exosomal immunosuppressive signal. Modified dendritic cell-derived exosomes (Dex) carrying miR-21 inhibitors could target GA-MSCs and reduce CD73 expression on MDSCs, synergizing with anti-PD-1 monoclonal antibody (mAb).

**Conclusions:**

Overall, this work reveals the critical role of MSCs in the glioma microenvironment as signal multipliers to enhance immunosuppressive signaling of glioma exosomes, and disrupting the positive feedback loop in MSCs with modified Dex could improve PD-1 blockade therapy.

**Graphical abstract:**

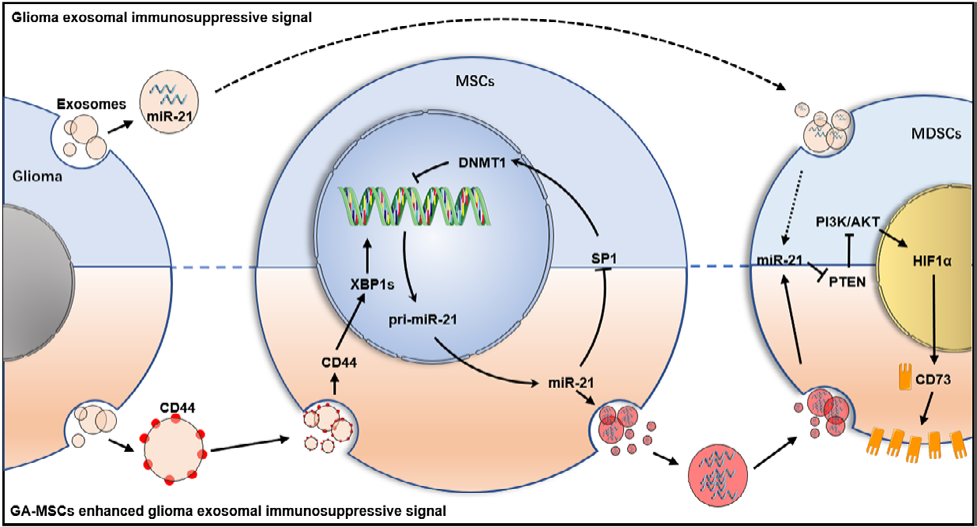

**Supplementary Information:**

The online version contains supplementary material available at 10.1186/s12951-023-01997-x.

## Introduction

Glioblastoma multiforme (GBM) is the most common primary malignant tumor of the central nervous system and is resistant to conventional therapies, including surgery, radiotherapy and chemotherapy [1, 2]. The median survival time of GBM patients receiving standard-of-care treatment is approximately 15 months [3]. In recent years, tumor immunotherapy has been applied in the treatment of various tumors, and satisfactory results have been obtained [4–6]. However, glioma is resistant to immunotherapies because of its unique immunosuppressive microenvironment.

Mesenchymal stem cells (MSCs) are a major component of the tumor microenvironment and are characterized by the coexpression of CD105, CD73, and CD90 [7, 8]. MSCs can modulate the immune response and have been reported to inhibit the proliferation of natural killer (NK) cells and promote the M2 polarization of macrophages [9, 10]. The percentage of MSCs in high-grade glioma was inversely correlated with patient survival, indicating that infiltrating MSCs could promote glioma progression [11], but the underlying mechanism remains unclear.

Myeloid-derived suppressor cells (MDSCs) play an important role in the formation of the glioma immunosuppressive microenvironment [12, 13]. Reportedly, MDSCs infiltration in glioma is positively correlated with malignant behavior [14]. MDSCs exhibit a CD11b^+^CD33^+^HLA-DR^−^ phenotype in humans and a CD11b^+^Gr-1^+^ phenotype in mice [15]. MDSCs can regulate the immune response through several immune-related molecules, such as CD73, Arg-1, and iNOS [16–18]. Inhibiting the immunosuppressive function of MDSCs is expected to improve the effect of immunotherapy in glioma and prolong patient survival time.

The ATP–adenosine pathway promotes the formation of an immunosuppressive microenvironment [19]. In the extracellular space, ATP or ADP can be converted into ADP or AMP, respectively, by the ectoenzyme CD39, and AMP can be converted into adenosine by the ectoenzyme CD73 [20]. Adenosine suppresses the functions of multiple immune cells, such as T cells, NK cells, and dendritic cells (DC), and promotes tumor progression [21]. An anti-CD73 monoclonal antibody (mAb) is currently being evaluated as a monotherapy targeting a variety of solid tumors in small-scale trials [22–24]. In addition, the absence of CD73 was shown to improve survival in a murine model of glioma treated with anti-PD-1 therapy, indicating that CD73 is a combination therapy target in glioma [25].

Exosomes are extracellular vesicles with a diameter of 30–150 nm that can be secreted from most cells [26]. Exosomes are mainly composed of lipid molecules, proteins and noncoding RNAs [27]. Our previous studies have shown that exosomes mediate communication between tumor cells and immune cells in the glioma microenvironment, promoting the malignant behavior of glioma cells and the formation of an immunosuppressive glioma microenvironment [28–30]. Studying the cargo of exosomes would be helpful for identifying new therapeutic strategies.

Noncoding RNAs play an important role in exosome-mediated communication [31]. It was reported that RNAs with a relatively shorter sequence are more easily loaded into exosomes and that the effect of exosomal noncoding RNA is dose dependent [31, 32]. MicroRNAs (miRNAs) are a class of 20- to 25-nucleotide noncoding RNAs [33]. Reportedly, miRNAs are the most enriched nucleotides in exosomes, implying an important role of miRNAs in exosome-mediated cell‒cell communication [34].

In this study, we found that glioma-associated mesenchymal stem cells (GA-MSCs) promoted glioma progression by upregulating CD73 expression on MDSCs through exosomal miR-21, which was similar to our previously reported finding that glioma-derived exosomal miR-21 promotes MDSC activation [35], but more intense. Here, we found that glioma-derived exosomal CD44 upregulated miR-21 expression in MSCs by stimulating the expression of the transcription factor XBP1s. Upregulated miR-21 in MSCs could reduce the DNA methylation level in the miR-21 promoter region through the miR-21/SP1/DNA methyltransferase 1 (DNMT1) pathway, further promoting the transcription of miR-21 and triggering a positive feedback loop. The miR-21/SP1/DNMT1 positive feedback loop in MSCs triggered by glioma exosomal CD44 could increase MSC exosomal miR-21 to a dozen times that in glioma exosomes, amplifying glioma exosomal immunosuppressive signaling. These results revealed the central role of MSCs in the glioma microenvironment as signal multipliers. On the basis of the above research, we designed miR-21 inhibitors containing exosomes modified with the blood‒brain barrier (BBB)-penetrating peptide angiopep-2 to disrupt the miR-21/SP1/DNMT1 positive feedback loop. Animal experiments demonstrated that modified exosomes could target GA-MSCs to inhibit glioma progression in vivo and synergize with anti-PD-1 mAb therapy.

## Materials and methods

### Cell culture

Mouse and human bone marrow-derived MSCs (BM-MSCs) were purchased from Cyagen Biosciences. Human glioma cell lines (U87MG and LN229) and the mouse glioma cell line GL261 were purchased from the Chinese Academy of Sciences Cell Bank. All cells were cultured in DMEM/F12 supplemented with 10% FBS, penicillin (100 U/ml), and streptomycin (100 U/ml). Human and mouse glioma-associated MSCs (GA-MSCs) were established by treating BM-MSCs with supernatant from U87MG or GL261 cells for 2 weeks. These cell lines were cultured in a humidified incubator containing 5% CO_2_ at 37 °C and validated by short tandem repeat profiling.

### Animal study

Four- to six-week-old male C57BL/6 mice were purchased from GemPharmatech Co., Ltd. (China) and maintained at the Neurosurgery Laboratory of Qilu Hospital of Shandong University. All experimental procedures were approved by the Animal Care and Use Committee of the Qilu Hospital of Shandong University.

To evaluate the glioma-promoting effect of MSCs in vivo, luciferase-expressing GL261 cells (5 × 10^5^/mouse) were mixed with mouse BM-MSCs or GA-MSCs (5 × 10^5^/mouse) and injected into the brains of C57BL/6 mice. Glioma growth was monitored using an IVIS spectrum in vivo imaging system (PerkinElmer) on days 4, 11, and 18 after glioma implantation. Mice were sacrificed after the last bioluminescence imaging to evaluate CD73 expression on MDSCs. Another 5 mice were used for survival analysis.

To assess the MDSC induction ability of MSC exosomes, normal C57BL/6 mice were intravenously injected with exosomes (30 µg/mouse/time) derived from mouse BM-MSCs or GA-MSCs three times a week. Two weeks later, the mice were sacrificed, and the splenocytes were collected for flow cytometry analysis.

For combined drug animal experiments, luciferase-expressing GL261 cells (10^6^/mouse) were injected into the brains of C57BL/6 mice. Seven days later, glioma-bearing mice were intravenously injected with modified mouse DC-derived exosomes (30 µg/mouse/time) containing miR-21 inhibitors three times a week for 2 weeks. Anti-PD-1 antibodies (BP0273, clone 29 F.1A12, BioXcell) and isotype controls (BP0089, BioXcell) were intraperitoneally injected into the mice (250 µg/mouse/time) on days 7, 10, 13, 16 and 19 after glioma implantation. Tumor volume was evaluated using bioluminescence imaging.

### Human MDSC induction

Lymphocyte separation medium (TBD, LTS1077) was used to isolate human peripheral blood mononuclear cells (PBMCs) from healthy volunteer venous blood following the manufacturer’s protocol. PBMCs collected from healthy volunteers were cultured in RPMI 1640 medium supplemented with 10% exosome-depleted FBS. Exosomes (10 µg) derived from human BM-MSCs or GA-MSCs were added to the culture medium 72 h before testing. Flow cytometry was used to determine the percentage of CD33^+^HLA-DR^−^ MDSCs and the expression of CD73 on MDSCs.

### Patient MDSCs isolation

CD33^+^HLA-DR^−^ MDSCs were separated from glioma patient-derived PBMCs using HLA-DR MicroBeads and CD33 MicroBeads (Miltenyi Biotec) following the manufacturer’s protocol. Ethics approval was obtained from the Clinical Research Ethics Committee of Qilu Hospital of Shandong University.

### Mouse MDSCs induction

Mouse bone marrow cells were cultured in RPMI 1640 medium supplemented with 10% exosome-depleted FBS and GM-CSF (20 ng/ml). Exosomes (10 µg) derived from mouse BM-MSCs or GA-MSCs were added to the culture medium 72 h before testing. Flow cytometry was used to determine the percentage of Gr-1^+^CD11b^+^ MDSCs and the expression of CD73 on MDSCs.

### Exosome isolation

Cell culture medium was collected and centrifuged at 2,000 × g for 30 min and 12,000 × g for 45 min, and the precipitate was removed. The supernatant was ultracentrifuged at 100,000 × g for 70 min. PBS was used to resuspend the deposited exosomes. All exosomes were stored in a -80 °C freezer, and repeated freezing and thawing was avoided.

### Small interfering RNA, miRNA mimics, and lentivirus transfection

All small interfering RNAs (siRNAs), miRNA mimics and inhibitors were purchased from GenePharma (Shanghai, China). Lipofectamine™ 3000 (Thermo Fisher, USA) was used to transfect siRNAs, miRNA mimics and inhibitors into cells according to the manufacturer’s protocol. The GNSTM-ANG-Lamp2B-HA plasmid (GenePharma, China) was constructed as we previously described [36]. All siRNA, mimic and inhibitor sequences are listed in Table S1.

### qRT–PCR and western blotting

For qRT‒PCR, total RNA was extracted from MSCs, MDSCs, and glioma cells using TRIzol in accordance with the manufacturer’s protocol. Exosomal RNA was extracted using the SeraMir™ Exosome RNA Extraction Kit (System Biosciences, USA) following the manufacturer’s protocol. A ReverTra Ace qPCR RT Kit (FSQ-101, TOYOBO) was used to perform reverse transcription. TB Green™ Premix Ex Taq™ (Takara) was used to conduct quantitative PCR. U6 and β-actin were used as internal controls for miRNA and mRNA, respectively. All primers used for qRT‒PCR are listed in Table S2.

For western blotting, protein was extracted from MSCs, MDSCs, glioma cells or their exosomes using RIPA lysis buffer (Beyotime, China) following the manufacturer’s protocol. All antibodies used for western blotting are listed in Table S3.

### T-cell suppression assay

To evaluate the capacity of mouse MDSCs to suppress T cells, single-cell suspensions of spleen or tumor tissue were stained with anti-Gr-1-FITC antibody for 10 min and anti-FITC microbeads (Miltenyi Biotec) for 15 min, and then the suspensions were loaded onto a magnetic column to separate the Gr-1^+^ cells. CFSE (2.5 µM; Invitrogen) was used to label splenocytes from normal C57BL/6 mice. After incubating splenocytes with CFSE for 20 min at 37 °C, the cells were stimulated with 2 µg/ml anti-mouse CD28 antibody in the culture medium (eBioscience, USA) and plate-bound anti-mouse CD3e antibody (eBioscience). Isolated Gr-1^+^ cells (1 × 10^5^) were cocultured with 2 × 10^5^ splenocytes labeled with CFSE in U-bottom 96-well plates. AMP (50 µM, Solarbio) was added to the culture medium. Three days later, cells were labeled with anti-mouse CD8-APC antibody, and CD8^+^ T-lymphocyte CFSE dilution was analyzed.

To assay the capacity of human MDSCs to suppress T cells, CD33-depleted PBMCs were labeled with 2.5 µM CFSE and stimulated with a plate-bound anti-human CD3 antibody (1 µg/ml, eBioscience) and an anti-human CD28 antibody (1 µg/ml, eBioscience) in the culture medium. Exosome-induced or miR-21 mimic-transfected CD33^+^ cells were cultured with these cells at a 1:2 ratio in U-bottom 96-well plates. AMP (50 µM) was added to the culture medium. Seventy-two hours later, the cells were stained with anti-human CD8-APC antibody, and CFSE dilution was analyzed.

### Methylation-specific PCR (MSP)

The QIAamp DNA Mini Kit (Qiagen) was used to extract genomic DNA from MSCs following the manufacturer’s protocol. Purified DNA was exposed to bisulfite using the EpiTect Bisulfite Kit (Qiagen). MSP of bisulfite-transformed DNA was carried out with a nested, two-stage PCR method. The primer sequences are listed in Table S2. The amplified PCR products were separated by agarose gel electrophoresis and visualized with GelRed.

### Luciferase reporter assay

Starbase (starbase.sysu.edu.cn/) was used to predict the targets of miR-21. Reporter genes containing pGL3-*DNMT1*-3’UTR, pGL3-*SP1*-1-wt, pGL3-*SP1*-2-wt, pGL3-*SP1*-1-mut, and pGL3-*SP1*-2-mut were synthesized (GeneChem, China). Jaspar (jaspar.genereg.net) was used to predict the binding site between XBP1s and the promoter of miR-21. Reporter genes containing pGL3-miR-21-promoter-wt and pGL3-miR-21-promoter-mut were synthesized (GeneChem, China). Lipofectamine™ 3000 reagent was used to transfect dual-luciferase reporter gene plasmids according to the manufacturer’s protocol. The Dual Luciferase Reporter Assay Kit (Promega, USA) was used to perform the luciferase assay 48 h after transfection.

### Flow cytometry

To measure CD73 expression on human MDSCs, human PBMCs were stained with anti-human CD73-APC (17–0739, eBioscience), anti-human CD33-FITC (11–0339, eBioscience) and anti-human HLA-DR-PE (12-9952, eBioscience) antibodies for 30 min at room temperature. To measure CD73 expression on mouse MDSCs, single cells from mouse bone marrow cells or spleen or tumor tissues were stained with anti-mouse CD73-APC (127,210, BioLegend), anti-mouse Gr-1-PE (108,408, BioLegend) and anti-mouse CD11b-FITC (11–0112, eBioscience) antibodies. A C6 flow cytometer (BD Biosciences, USA) was used to perform flow cytometry. FlowJo V10 software was used to analyze the data.

To measure DNMT1 expression in MSCs, FoxP3/Transcription Factor Staining Buffer Kit (IC001, Multi Sciences) was used to permeabilize cells according to the manufacturer’s protocol. Anti-mouse CD45-FITC (11–0454, eBioscience), Anti-mouse TER-119-FITC (11-5921, eBioscience), Anti-mouse Sca-1-PE-Cyanine7 (25-5981) and anti-mouse PDGFRA-APC (17-1401, eBioscience) were used to identify MSCs. Anti-Dnmt1 antibody (ab188453, Abcam) and PE–conjugated Goat Anti-Rabbit IgG (H + L) (SA00008-2, Proteintech) were used to measure DNMT1 expression.

Prior to IFN-γ staining, cells were stimulated with 50 ng/mL PMA (Sigma), 750 ng/mL ionomycin (Sigma) and 1×GolgiPlug™ (BD Biosciences) at 37 °C for 4 h. Then the FoxP3/Transcription Factor Staining Buffer Kit, anti-mouse CD8-APC (17–0081, eBioscience) and anti-mouse IFN-γ-PE (F21IFNG02, Multi Sciences) was used to evaluate the percentage of CD8^+^IFN-γ^+^ cells in glioma tissues.

### Chromatin immunoprecipitation (ChIP)

A ChIP Assay Kit (Beyotime, China) was used to precipitate DNA combined with anti-XBP1s antibody (40,435 S, Cell Signaling Technology) according to the manufacturer’s protocol. A DNA Cleanup Kit (Beyotime, China) was used to purify DNA precipitated by the ChIP Assay Kit. The ChIP-specific primers used for qRT‒PCR are listed in Table S2.

### Dendritic cell-derived exosome (dex) generation

Dendritic cells were generated from C57BL/6 mice by culturing bone marrow cells at 1 × 10^6^ cells/ml in RPMI 1640 medium supplemented with 10% exosome-depleted FBS, GM-CSF (20 ng/ml) and IL-4 (20 ng/ml) [37]. Four days later, cells were transfected with the GNSTM-ANG-Lamp2B-HA plasmid. The cell culture medium was replaced with fresh culture medium on day 6. Two days later, the culture medium was collected for exosome isolation.

### Exosome loading

miR-21 inhibitors were loaded into ANG-modified mouse Dex by electroporation using a single 4 mm cuvette and a Lonza Nucleofector 2B system as we previously described [36].

### Statistical analysis

GraphPad software 9 was used to analyze the data. Student’s t test was used to analyze comparisons between two groups. One-way ANOVA was used to analyze comparisons between multiple groups. Survival curves were generated using the Kaplan‒Meier method and compared using the log-rank test. All data are presented as the mean ± standard deviation. The significance of differences between different groups is marked in the figures (*P < 0.05, **P < 0.01, and ***P < 0.001).

## Results

### GA-MSCs promote glioma progression by inducing CD73 expression on MDSCs

**Fig. 1:**
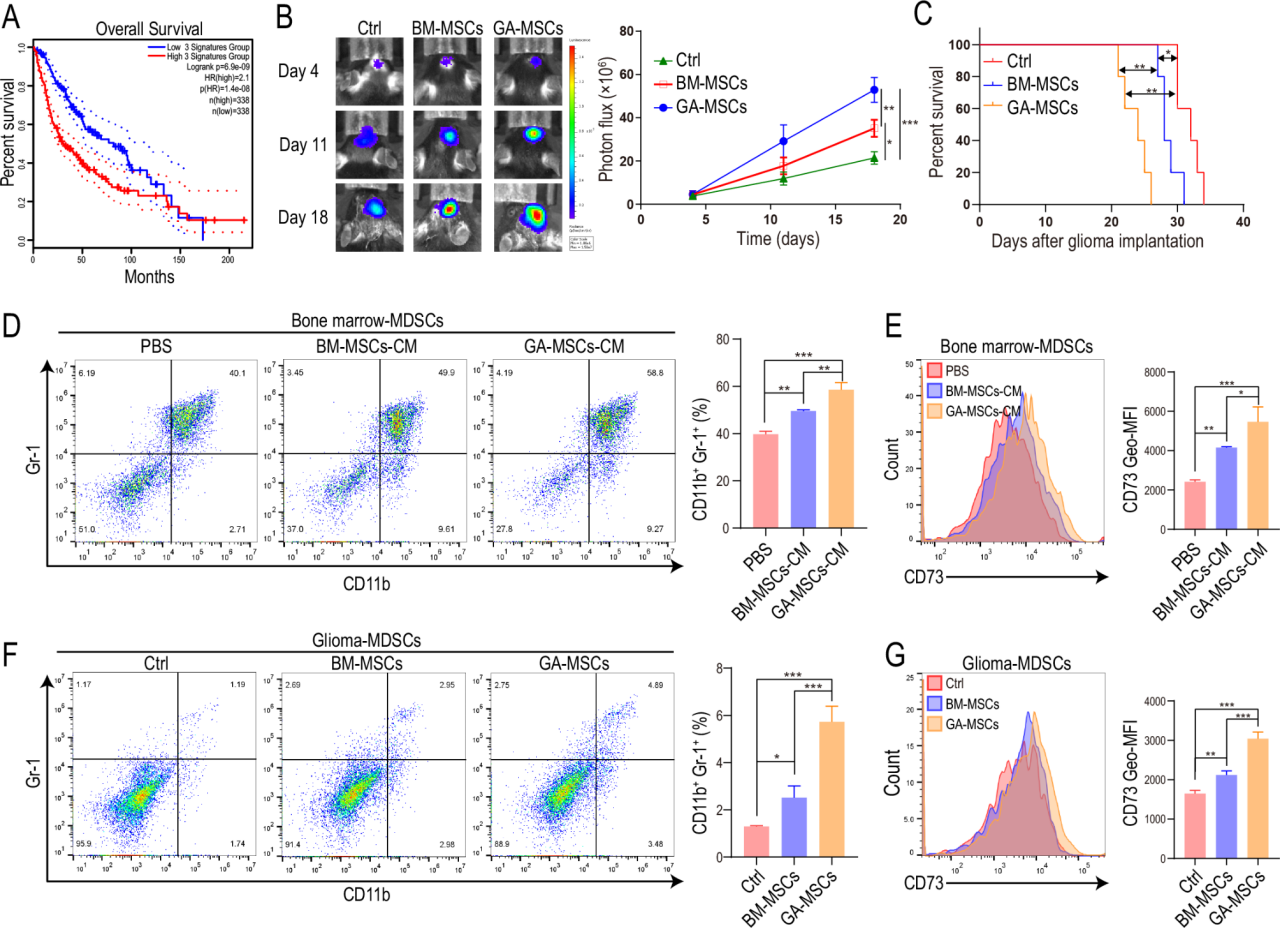
GA-MSCs promoted glioma growth by upregulating CD73 expression on MDSCs **(A)** Survival curves of glioma patients with high (upper 50% of gene expression) and low (lower 50% of gene expression) levels of CD73, CD90, and CD105 expression based on data from TCGA. **(B, C)** Representative bioluminescence images, luminescence quantification (n = 3 for each group) and survival analysis (n = 5 for each group) for mice implanted with GL261 cells (Ctrl group), GL261 cells mixed with bone marrow MSCs (BM-MSCs groups) or GL261 cells mixed with glioma-associated MSCs (GA-MSCs group). **(D, E)** PBS, mouse BM-MSCs-derived culture medium (CM) or GA-MSCs-derived CM was used to stimulate mouse bone marrow cells. The percentage of Gr-1^+^CD11b^+^ MDSCs and the expression of CD73 on MDSCs were measured by flow cytometry. **(F, G)** The percentage of Gr-1^+^CD11b^+^ MDSCs and the expression of CD73 on MDSCs infiltrating in glioma tissues in mice in (B) were measured by flow cytometry. The data are presented as the mean ± SD; *p < 0.05, **p < 0.01, and ***p < 0.001

To clarify the role of MSCs in glioma progression, we classified glioma patients in The Cancer Genome Atlas (TCGA) into MSC-high and MSC-low groups based on coexpression of the MSC markers CD73, CD90 and CD105 (accessed via http://gepia2.cancer-pku.cn/). Patients in the MSC-high group exhibited significantly shorter overall survival (Fig. 1A). To investigate the function of GA-MSCs in vivo, mouse bone marrow MSCs (BM-MSCs) or GA-MSCs (induced with conditioned medium (CM) from the mouse glioma cell line GL261) were coimplanted with GL261 cells into C57BL/6 mice in situ. The control group consisted of mice implanted with GL261 cells alone. We found that BM-MSCs promoted the growth of glioma and reduced the survival time of mice and that GA-MSCs had a stronger glioma-promoting effect than BM-MSCs (Fig. 1B, C).

To study the mechanism of the GA-MSCs-mediated glioma-promoting effect, CM from BM-MSCs or GA-MSCs was used to treat GL261 cells, and we found that both BM-MSCs CM and GA-MSCs CM promoted GL261 cell proliferation (Fig. S1A), indicating that the poor prognosis caused by MSCs might be due to promotion of glioma cell proliferation. However, there was no significant difference between BM-MSC CM and GA-MSC CM in promoting GL261 cell proliferation (Fig. S1A). Considering that GA-MSCs promoted glioma progression more significantly than BM-MSCs (Fig. 1B-C), there must be another mechanism responsible for the poor prognosis caused by GA-MSCs. BM-MSC CM and GA-MSC CM were further used to stimulate CD8^+^ T cells and bone marrow cells. No significant difference was observed in the inhibition of CD8^+^ T-cell proliferation between BM-MSC CM and GA-MSC CM (Fig. S1B), though CM from GA-MSCs had a stronger ability to induce the differentiation of CD11b^+^Gr-1^+^ MDSCs (Fig. 1D), implying that GA-MSCs might promote glioma progression by inducing MDSCs and promoting the formation of an immunosuppressive microenvironment. By performing qRT‒PCR, we found that both BM-MSC CM and GA-MSC CM promoted *Arg-1* and *iNOS* expression in MDSCs, but there was no significant difference between them (Fig. S1C). MDSCs have been reported to suppress T-cell function by expressing CD73 [38], a cell-surface marker of MSCs, which led us to test whether GA-MSCs could strengthen the suppressive function of MDSCs against T cells by inducing CD73 expression. We found that GA-MSCs had a stronger ability to induce CD73 expression on CD11b^+^Gr-1^+^ MDSCs than BM-MSCs (Fig. 1E).

To validate the MDSC induction ability of GA-MSCs in vivo, mouse BM-MSCs or GA-MSCs were coimplanted with GL261 cells into C57BL/6 mice in situ. We found that GA-MSCs promoted the infiltration of CD11b^+^Gr-1^+^ MDSCs in glioma (Fig. 1F), and CD73 expression on MDSCs was higher in the GA-MSC group than in the BM-MSC group and control group (Fig. 1G). Furthermore, MDSCs derived from the GA-MSC group had the strongest ability to suppress T-cell proliferation, and the T-cell-suppressive function of MDSCs could be inhibited by a CD73 inhibitor (M8386, Sigma‒Aldrich) (Fig. S1D), indicating that GA-MSCs promoted the formation of an immunosuppressive microenvironment in glioma by upregulating CD73 expression on MDSCs.

### GA-MSC-derived exosomes increase CD73 expression on MDSCs

**Fig. 2:**
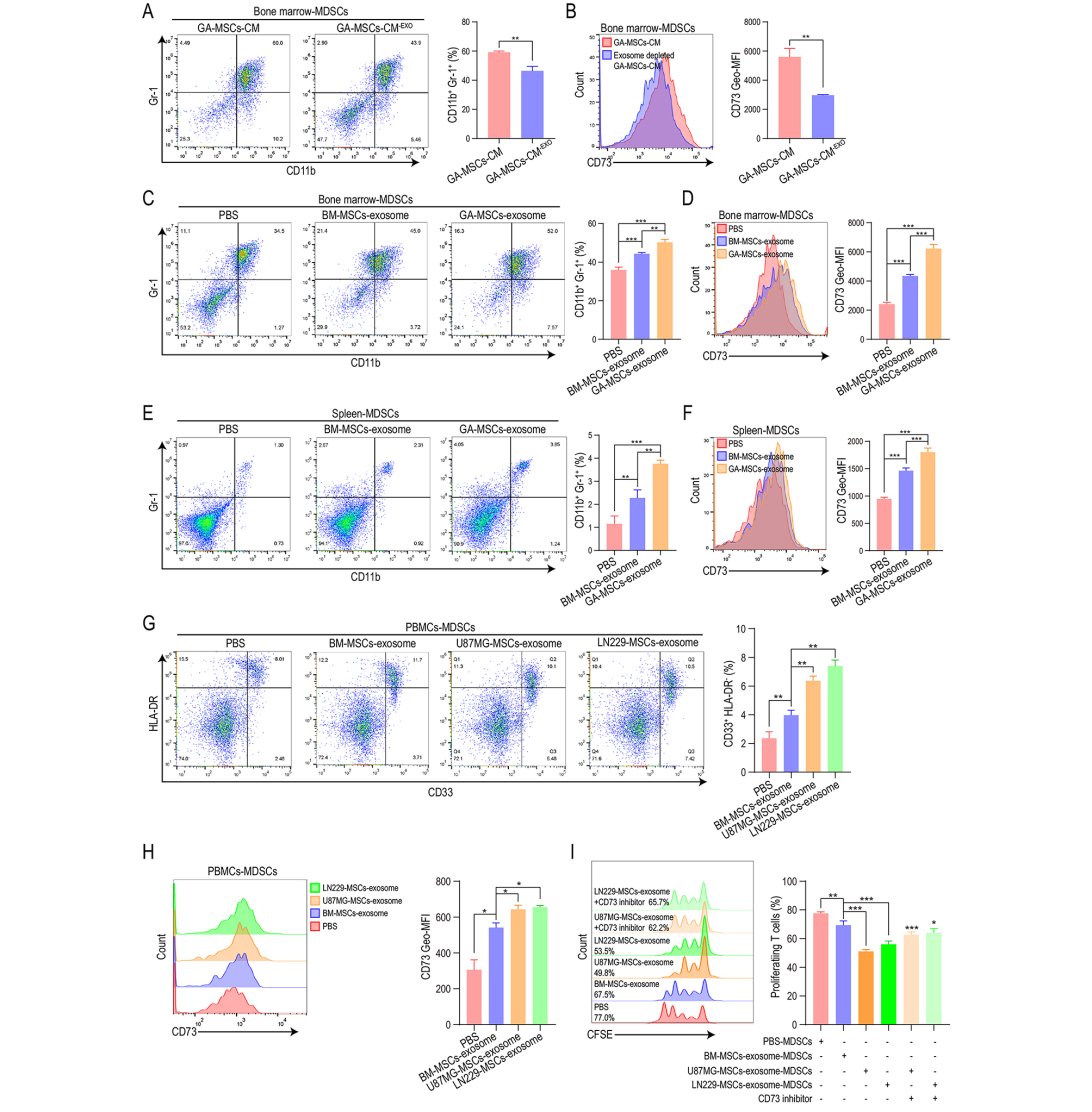
GA-MSC-derived exosomes increase CD73 expression on MDSCs **(A, B)** Mouse GA-MSCs-CM and GA-MSCs-CM^− EXO^ (GA-MSCs-CM depleted of exosomes) were used to stimulate mouse bone marrow cells. The percentage of Gr-1^+^CD11b^+^ MDSCs and the expression of CD73 on MDSCs were measured by flow cytometry. **(C, D)** PBS, mouse BM-MSCs-derived exosomes or GA-MSCs-derived exosomes were used to stimulate mouse bone marrow cells. The percentage of Gr-1^+^CD11b^+^ MDSCs and the expression of CD73 on MDSCs were measured. **(E, F)** PBS, mouse BM-MSCs-derived exosomes or GA-MSCs-derived exosomes were intravenously injected into mice. The percentage of Gr-1^+^CD11b^+^ splenic MDSCs and the expression of CD73 on splenic MDSCs were measured. **(G, H)** Exosomes were isolated from the culture medium of human BM-MSCs and GA-MSCs (induced by U87MG and LN229 cell supernatant) and used to treat PBMCs. The percentage of CD33^+^HLA-DR^−^ MDSCs and the expression of CD73 on MDSCs were measured by flow cytometry. **(I)** Exosome-induced PBMCs-derived MDSCs were cocultured with CFSE-labeled CD33-depleted PBMCs in the absence or presence of CD73 inhibitor. After 72 h of coculture, CD8^+^ T-cell proliferation was measured using flow cytometry. The data are presented as the mean ± SD; *p < 0.05, **p < 0.01, and ***p < 0.001

Exosomes play an important role in cell‒cell communication and the formation of the immunosuppressive glioma microenvironment [39]. To investigate whether GA-MSCs promote CD73 expression on MDSCs through exosomes, mouse GA-MSC-derived CM was depleted of exosomes by ultracentrifugation and used to stimulate mouse bone marrow cells. We found that depleting exosomes inhibited the MDSC induction ability of GA-MSC CM (Fig. 2A, B). Furthermore, we stimulated mouse bone marrow cells with exosomes derived from mouse BM-MSCs or GA-MSCs and found that the GA-MSC-derived exosomes had a stronger ability to increase CD73 expression on MDSCs (Fig. 2C, D). To validate the MDSC induction function of exosomes in vivo, mice were injected with BM-MSCs- or GA-MSCs-derived exosomes via the tail vein. We found that GA-MSCs-derived exosomes induced the highest level of CD73 expression on splenic MDSCs (Fig. 2E, F). Splenic MDSCs induced by GA-MSCs-derived exosomes also showed the strongest T-cell-suppressive ability, which could be inhibited by a CD73 inhibitor (Fig. S2A). These results indicated that mouse GA-MSCs increased CD73 expression on MDSCs through exosomes both in vitro and in vivo.

In addition, exosomes were isolated from the CM of human BM-MSCs and GA-MSCs (induced with CM from the human glioma cell line U87MG or LN229) and used to stimulate human PBMCs. BM-MSCs-derived exosomes significantly promoted the differentiation of CD33^+^HLA-DR^−^ MDSCs, and GA-MSCs-derived exosomes exhibited a stronger MDSCs induction capacity than BM-MSCs-derived exosomes (Fig. 2G). We also measured CD73 expression on PBMCs-derived MDSCs and found that GA-MSC-derived exosomes induced the most significant upregulation of CD73 expression (Fig. 2H). GA-MSCs-derived exosome-induced MDSCs also had the strongest ability to suppress T-cell proliferation, and the CD73 inhibitor impaired the T-cell-suppressive ability of GA-MSC-derived exosome-induced MDSCs (Fig. 2I). These findings suggest that human GA-MSCs could also increase CD73 expression on MDSCs through exosomes.

### GA-MSCs-derived exosomal miR-21 promoted CD73 expression through the PTEN/PI3K/AKT/HIF-1α pathway

**Fig. 3:**
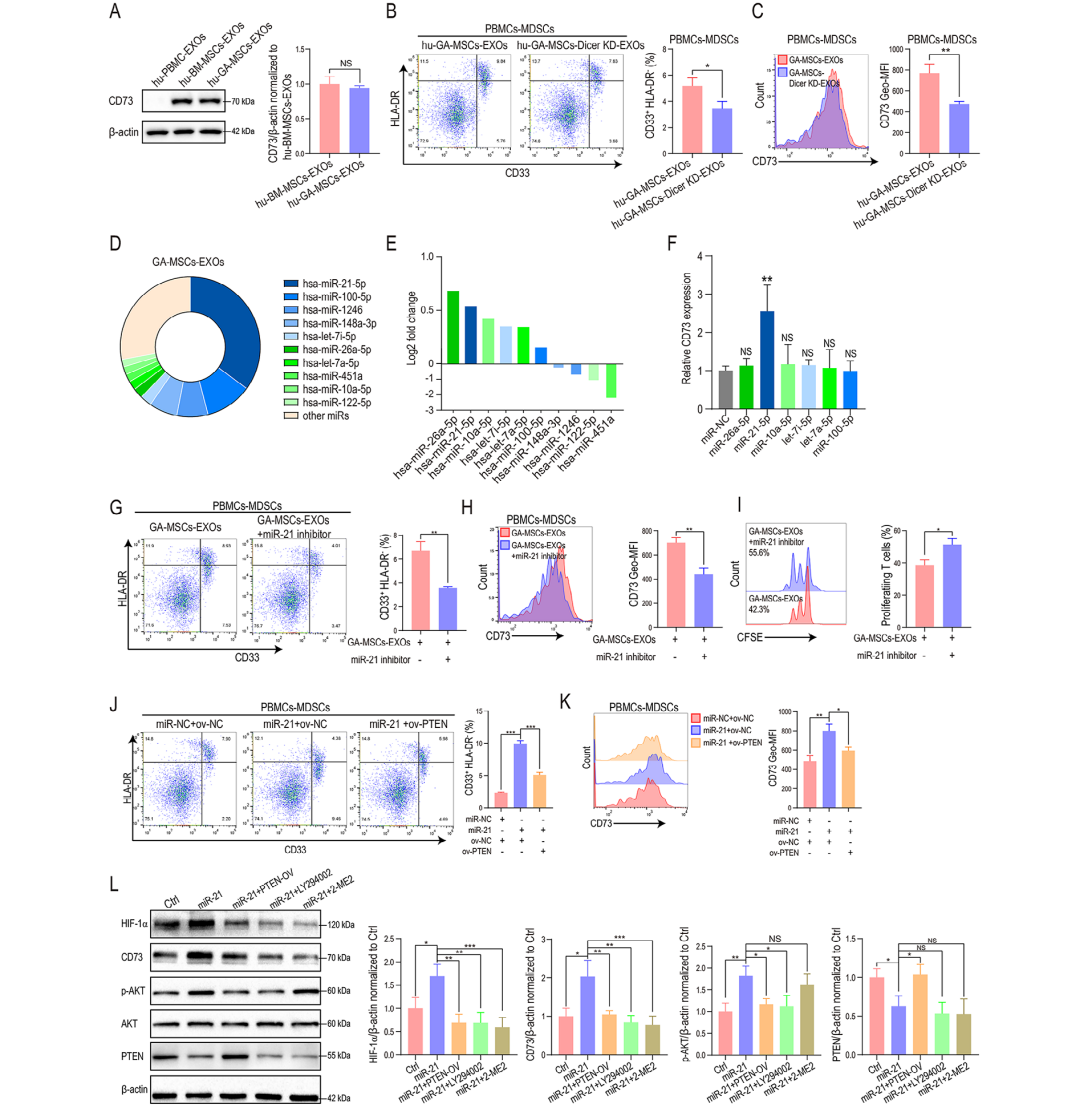
Human GA-MSCs-derived exosomal miR-21 upregulated CD73 expression on MDSCs through the PTEN/PI3K/AKT/HIF-1α pathway **(A)** CD73 expression in hu-BM-MSCs-EXOs (exosomes derived from human BM-MSCs) and hu-GA-MSCs-EXOs (exosomes derived from human GA-MSCs) was measured using western blotting. PBMC-derived exosomes were used as a negative control. Quantification of the fold change in the CD73/β-actin ratio (normalized to hu-BM-MSCs-EXOs) is shown. **(B, C)** Exosomes derived from human GA-MSCs with or without *DICER* knockdown were used to stimulate human PBMCs. The percentage of CD33^+^HLA-DR^−^ MDSCs and the expression of CD73 on MDSCs were measured by flow cytometry. **(D)** Distribution of the top 10 most highly expressed miRNAs in human GA-MSCs-derived exosomes. **(E)** The ratio of GA-MSCs-derived exosome intensity versus BM-MSCs-derived exosome intensity is presented for the top 10 most highly expressed miRNAs. **(F)** The upregulated miRNAs in the top 10 most highly expressed miRNAs were transfected into glioma patient-derived CD33^+^HLA-DR^−^ MDSCs. The expression of CD73 was measured using qRT‒PCR. **(G, H)** PBMCs were stimulated with human GA-MSCs-derived exosomes and transfected with miR-21 inhibitor. The percentage of CD33^+^HLA-DR^−^ MDSCs and the expression of CD73 on MDSCs were measured by flow cytometry. **(I)** MDSCs obtained by stimulating CD33^+^ cells with GA-MSCs-derived exosomes were transfected with miR-21 inhibitor. CD33-depleted PBMCs were labeled with CFSE and cocultured with MDSCs for 72 h. CD8^+^ T-cell proliferation was measured by flow cytometry **(J, K).** The percentage of MDSCs and CD73 expression on MDSCs induced by miR-NC or miR-21 mimics and nonsense sequence or PTEN overexpression plasmids were measured using flow cytometry. **(L)** The expression of HIF-1α, CD73, p-AKT and PTEN was measured in miR-21-overexpressing MDSCs transfected with PTEN overexpression plasmid or treated with a PI3K inhibitor (LY294002) or HIF-1α inhibitor (2-ME2). Quantification of the fold change in the HIF-1α/β-actin, CD73/β-actin, p-AKT/β-actin and PTEN/β-actin ratios (normalized to Ctrl) are shown. The data are presented as the mean ± SD; *p < 0.05, **p < 0.01, and ***p < 0.001

To investigate whether the upregulation of CD73 on MDSCs induced by GA-MSC-derived exosomes was caused by direct transfer of the CD73 protein, we measured CD73 protein expression in exosomes derived from human and mouse BM-MSCs and GA-MSCs. There was no significant upregulation of CD73 in GA-MSC-derived exosomes compared to BM-MSC-derived exosomes (Fig. 3A, Fig. S3A), indicating that the stronger CD73 induction ability of GA-MSCs-derived exosomes was not a result of direct transfer of the CD73 protein. To further confirm that exosomal miRNA is responsible for the upregulation of CD73, we knocked down *DICER* expression in GA-MSCs. Reportedly, Dicer is required for the maturation of miRNA, and inhibiting Dicer dramatically reduces miRNA expression in exosomes [40]. We found that *DICER* knockdown impaired the CD73 induction ability of both human and mouse GA-MSCs-derived exosomes (Fig. 3B, C; Fig. S3B, C), indicating that GA-MSCs-derived exosomes induce CD73 expression on MDSCs in an miRNA-dependent manner.

To identify the exosomal miRNAs responsible for CD73 upregulation, we performed miRNA sequencing of exosomes derived from human bone marrow MSCs (hu-BM-MSCs) and hu-GA-MSCs (induced with CM from U87MG cells) (SRA no. PRJNA814416). The top 10 most highly expressed miRNAs accounted for > 70% of the total miRNAs in hu-GA-MSCs-derived exosomes (Fig. 3D). Among the top 10 miRNAs highly expressed in GA-MSCs-derived exosomes, six were upregulated compared to BM-MSCs-derived exosomes (Fig. 3E). We transfected these upregulated miRNAs into CD33^+^HLA-DR^−^ MDSCs derived from glioma patients and measured CD73 expression. qRT‒PCR assays revealed that only miR-21 significantly upregulated CD73 expression (Fig. 3F). miR-21 was also the most abundant miRNA in the exosomes derived from GA-MSCs, accounting for 35% of the total miRNA content (Fig. 3D). qRT‒PCR confirmed that miR-21 was upregulated in both human and mouse GA-MSCs-derived exosomes compared with BM-MSC-derived exosomes (Fig. S3D). To further validate the function of miR-21, we transfected human PBMCs or mouse bone marrow cells with miR-21 inhibitor and stimulated them with human or mouse GA-MSC-derived exosomes, respectively. The miR-21 inhibitor impaired GA-MSCs-derived exosome-induced MDSC differentiation, CD73 expression, and immunosuppressive function (Fig. 3G-I; Fig. S3E, F).

Previously, we reported that miR-21 promoted the differentiation and activation of mouse MDSCs by targeting PTEN [35], but whether miR-21 could promote the differentiation of human MDSCs and increase CD73 expression on MDSCs remained unknown. We found that overexpression of PTEN reversed the MDSC differentiation-inducing and CD73-upregulating effects of miR-21 on human MDSCs (Fig. 3J, K). Similar phenomena were observed for mouse MDSCs (Fig. S3G, H). HIF-1α is the most important transcription factor regulating CD73 expression in the tumor microenvironment [38, 41]. Our previous study demonstrated that miR-21 could promote the differentiation and activation of MDSCs by activating the PTEN/PI3K/AKT pathway [35], and several studies have reported that the PI3K/AKT pathway can regulate the transcription and protein stability of HIF-1α [42, 43], so we speculated that miR-21 can regulate the expression of CD73 through the PTEN/PI3K/AKT/HIF-1α pathway. We found that PTEN overexpression blocked the upregulation of CD73 and HIF-1α in miR-21-overexpressing MDSCs (Fig. 3L). Moreover, a PI3K inhibitor (LY294002, Beyotime) and HIF-1α inhibitor (2-ME2, MedChemExpress) also inhibited the expression of CD73 in miR-21-overexpressing MDSCs (Fig. 3L), demonstrating that miR-21 promoted CD73 expression through the PTEN/PI3K/AKT/HIF-1α pathway.

In our previous study, we found that glioma cells also promoted the immunosuppressive function of MDSCs through exosomal miR-21 [35]. Our miRNA sequencing results showed that the content of miR-21 in glioma exosomes accounted for only 7% of the total miRNA content (Fig. S3I), while the content of miR-21 in GA-MSCs exosomes accounted for 35% of the total miRNA content (Fig. 3D). We further compared the expression of miR-21 in exosomes derived from human and mouse glioma cells and GA-MSCs, using the nematode miRNA cel-miR-39 as an external reference, and found that miR-21 expression in both human and mouse GA-MSC-derived exosomes was significantly higher than that in glioma-derived exosomes (Fig. S3J), indicating the important role of GA-MSCs in mediating the formation of the exosomal miR-21-induced immunosuppressive microenvironment.

### Upregulation of the transcription factor XBP1s induced by glioma exosomal CD44 promoted miR-21 expression in GA-MSCs

**Fig. 4:**
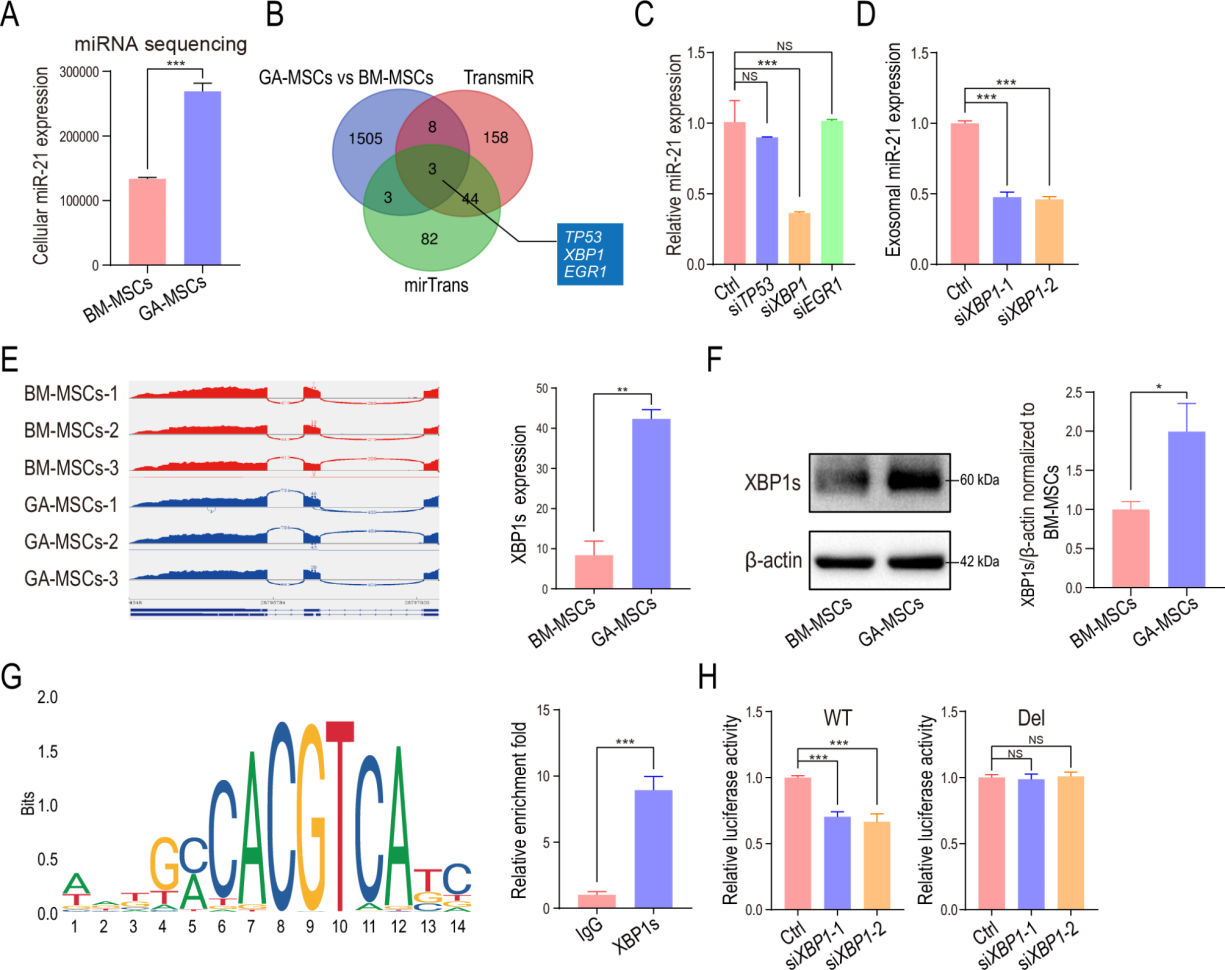
Upregulation of the transcription factor XBP1s in GA-MSCs promoted miR-21 expression **(A)** miR-21 expression in human BM-MSCs and GA-MSCs was measured using miRNA sequencing. **(B)** The upregulated genes in GA-MSCs were filtered by overlapping them with predicted miR-21 transcription factors (determined with the transmiR and mirTrans databases). **(C)** The expression of miR-21 was measured in human BM-MSCs with *TP53*, *XBP1*, or *EGR1* knockdown. **(D)** miR-21 expression was measured in control and *XBP1* knockdown human BM-MSCs-derived exosomes. **(E)** Transcriptome variable shear analysis showed the expression of XBP1s in human BM-MSCs and GA-MSCs. **(F)** XBP1s expression in human BM-MSCs and GA-MSCs was measured using western blotting. Quantification of the fold change in the XBP1s/β-actin ratio (normalized to BM-MSCs) is shown. **(G)** The XBP1 binding site in the miR-21 promoter region was predicted using Jaspar, and ChIP‒qPCR was used to confirm the binding in BM-MSCs. **(H)** The luciferase activity of the wild-type and mutant-type plasmids in human BM-MSCs transfected with si-NC or si-*XBP1* was measured. The data are presented as the mean ± SD; *p < 0.05, **p < 0.01, and ***p < 0.001

To investigate the underlying mechanism of exosomal miR-21 upregulation in GA-MSCs, we performed miRNA sequencing (SRA no. PRJNA814429) and transcriptome sequencing (SRA no. PRJNA816564) of human GA-MSCs and BM-MSCs and found that miR-21 expression was significantly increased in GA-MSCs compared to BM-MSCs (Fig. 4A), indicating that the elevated exosomal miR-21 expression was caused by the upregulation of cellular miR-21. qRT‒PCR results also confirmed that the expression of miR-21 and pri-miR-21 were higher in GA-MSCs than in BM-MSCs (Fig. S4A), indicating that the transcription of miR-21 was increased in GA-MSCs. The increased miRNA transcription could be caused by the upregulation of transcription factors and downregulation of DNA methylation in the promoter region. We next aimed to identify whether there was a key transcription factor responsible for upregulating miR-21. The upregulated genes in GA-MSCs were filtered by overlapping them with predicted miR-21 transcription factors (determined with the TransmiR and mirTrans databases). TP53, XBP1, and EGR1 were identified as potential transcription factors of miR-21 (Fig. 4B). We further validated their functions by knocking down these 3 genes in human BM-MSCs. *XBP1* knockdown reduced the expression of miR-21 and pri-miR-21 in BM-MSCs (Fig. 4C, Fig. S4B) as well as that of miR-21 in exosomes (Fig. 4D), indicating that XBP1 might be a transcription factor regulating miR-21.

XBP1 is an endoplasmic reticulum (ER) stress response factor. During ER stress, IRE1α cleaves the XBP1 mRNA transcript into a spliced form that encodes the active transcription factor XBP1s [44]. Our transcriptome variable shear analysis showed that the XBP1s level was increased significantly in GA-MSCs (Fig. 4E), which was confirmed by western blotting (Fig. 4F). To further investigate whether XBP1s is the transcription factor that regulates miR-21, the XBP1 binding site in the promoter of miR-21 was predicted using the JASPAR database and validated using a ChIP assay. A high binding affinity for XBP1s to the miR-21 promoter region was observed (Fig. 4G). Next, wild-type and mutant-type (predicted binding site deletion) luciferase reporters containing the miR-21 promoter were engineered and transfected into *XBP1* knockdown BM-MSCs. *XBP1* knockdown reduced the luciferase activity of the wild-type promoter but had no effect on the mutant-type promoter, further demonstrating that XBP1s promotes miR-21 transcription by binding to the predicted binding site in the miR-21 promoter (Fig. 4H).

We next sought to determine the underlying mechanism of XBP1s upregulation in GA-MSCs and whether glioma exosomes play a regulatory role in this process. We found that glioma-derived exosomes (GDEs) increased XBP1s expression in human BM-MSCs and that exosomes isolated from *DICER* knockdown glioma (GDEs-DICER-KD) still increased XBP1s expression in BM-MSCs, suggesting that glioma exosomes can upregulate XBP1s expression in human BM-MSCs in an miRNA-independent manner (Fig. S4C).

We treated BM-MSCs with exosomes derived from U87MG cells or human BM-MSCs and found that the U87MG-derived exosomes promoted the upregulation of XBP1s, while the BM-MSCs-derived exosomes did not (Fig. S4D), implying that some factors highly expressed in the U87MG-derived exosomes but expressed at low levels in the BM-MSC-derived exosomes might be responsible for XBP1s upregulation. We analyzed the exosomal protein profile data of U87MG and BM-MSCs [45] and screened for proteins that were highly expressed in U87MG cell-derived exosomes but hardly expressed in BM-MSC-derived exosomes (Fig. S4E). XBP1s is upregulated during ER stress and unfolded protein response (UPR) activation [46]. By analyzing our transcriptome sequencing data using gene set enrichment analysis (GSEA), we found that GA-MSCs were more positively correlated with ER stress and UPR activation than BM-MSCs (Fig. S4F). Among the highly expressed proteins screened in Figure S4E, CD44 was positively correlated with ER stress and UPR activation (Fig. S4G), and the expression of XBP1 was positively correlated with CD44 expression in glioma data from TCGA (Fig. S4H), indicating that glioma exosomal CD44 might be responsible for ER stress and XBP1s upregulation in MSCs. Western blot results confirmed that the CD44 expression in U87MG-derived exosomes was significantly higher than that in BM-MSCs-derived exosomes (Fig. S4I). In addition, we found the CD44 expression in U87MG was higher than that in BM-MSC and normal human brain tissues (Fig. S4J). Recombinant CD44 protein treatment significantly increased XBP1s expression in BM-MSCs (Fig. S4K), and promoted the expression of miR-21 and pri-miR-21 in human BM-MSCs, as well as that of miR-21 in BM-MSC-derived exosomes (Fig. S4L, M). In addition, knocking down *CD44* in glioma exosomes impaired the ability of glioma exosomes to induce XBP1s and miR-21 expression in BM-MSCs (Fig. S4N), indicating that glioma exosomal CD44 promoted XBP1s expression and upregulated cellular and exosomal miR-21 expression in MSCs.

### The miR-21/SP1/DNMT1 positive feedback loop in MSCs promoted miR-21 transcription

**Fig. 5:**
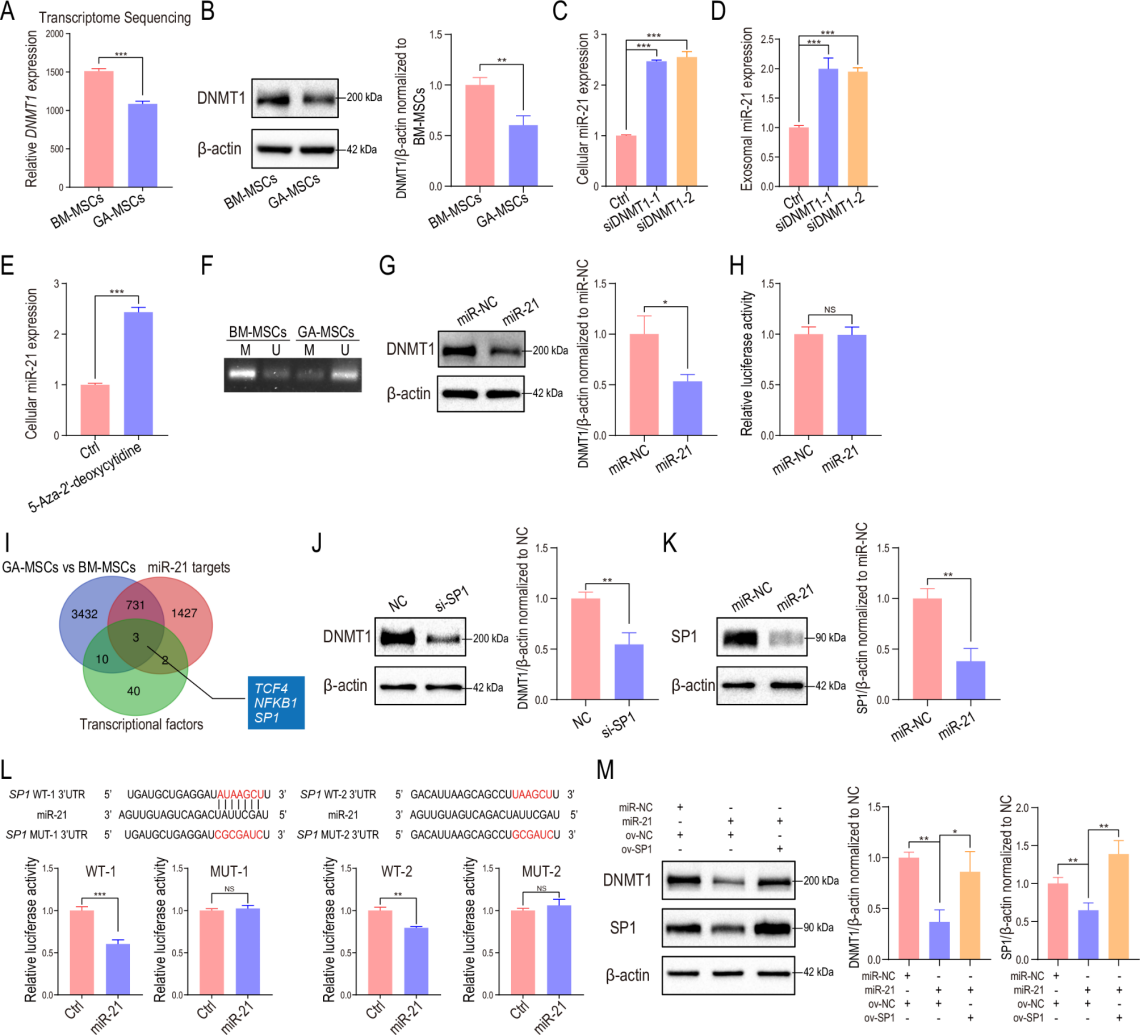
The miR-21/SP1/DNMT1 positive feedback loop in MSCs promoted miR-21 expression **(A)***DNMT1* expression in human BM-MSCs and GA-MSCs was measured using mRNA sequencing. **(B)** DNMT1 expression in human BM-MSCs and GA-MSCs was measured using western blotting. Quantification of the fold change in the DNMT1/β-actin ratio (normalized to BM-MSCs) is shown. **(C)** miR-21 expression was measured in control and *DNMT1* knockdown human BM-MSCs. **(D)** miR-21 expression was measured in control and *DNMT1* knockdown human BM-MSCs-derived exosomes. **(E)** miR-21 expression was measured in BM-MSCs treated with the DNA methylation inhibitor 5-aza-2’-deoxycytidine. **(F)** MSP analysis was performed to examine the methylation status of CpG islands in the promoter region of miR-21 in BM-MSCs and GA-MSCs. **(G)** DNMT1 expression in human BM-MSCs transfected with miR-21 mimics was measured using western blotting. Quantification of the fold change in the DNMT1/β-actin ratio (normalized to NC) is shown. **(H)** BM-MSCs were cotransfected with miR-21 and a luciferase reporter containing the 3’UTR of *DNMT1*. The luciferase activity was measured. **(I)** The downregulated genes in GA-MSCs were filtered by overlapping them with predicted miR-21 targets (determined with starBase) and predicted DNMT1 transcription factors (determined with the PROMO database). **(J)** DNMT1 expression was measured in control and *SP1* knockdown human BM-MSCs. Quantification of the fold change in the DNMT1/β-actin ratio (normalized to NC) is shown. **(K)** SP1 expression was measured in control and miR-21-overexpressing human BM-MSCs. Quantification of the fold change in the SP1/β-actin ratio (normalized to miR-NC) is shown. **(L)** Construction of wild-type (WT) and mutant-type (MUT) luciferase reporter vectors based on the predicted binding site of miR-21 in *SP1.***(M)** The expression of DNMT1 in MSCs transfected with miR-NC or miR-21 mimics and nonsense sequence or SP1 overexpression plasmids was measured using western blotting. The data are presented as the mean ± SD; *p < 0.05, **p < 0.01, ***p < 0.001

In addition to upregulation of transcription factors, altered DNA methylation in the promoter region also leads to upregulation of miRNA expression [47]. Our transcriptome sequencing and western blot results showed that the expression of DNMT1 was reduced in human GA-MSCs compared to BM-MSCs (Fig. 5A, B), and we found that *DNMT1* knockdown increased miR-21 expression in both human BM-MSCs and exosomes (Fig. 5C, D), indicating that the reduced DNA methylation level was responsible for the upregulated miR-21 expression. Similar results were observed for mouse MSCs (Fig. S5A-C). The Gene Expression Omnibus (GEO) datasets GSE124879 and GSE109273 were analyzed to further confirm that reduced DNA methylation was associated with increased miR-21 expression (Fig. S5D). In addition, we found that the DNA methylation inhibitor 5-aza-2’-deoxycytidine upregulated cellular miR-21 expression in human BM-MSCs (Fig. 5E). To further demonstrate the effect of DNA methylation on miR-21 expression, we used MethPrimer (http://www.urogene.org/cgi-bin/methprimer/methprimer.cgi) to predict CpG islands in the promoter region of miR-21 (Fig. S5E). A specific primer set was designed to detect changes in the DNA methylation levels of CpG islands in BM-MSCs and GA-MSCs. The DNA methylation levels in the miR-21 promoter region were decreased in GA-MSCs compared to BM-MSCs (Fig. 5F).

Interestingly, it has been reported that miR-21 can downregulate DNMT1 expression, although the underlying mechanism is unclear [48]. Western blot results confirmed that miR-21 overexpression downregulated DNMT1 expression in human BM-MSCs (Fig. 5G). Considering that no binding site for miR-21 was predicted in the human *DNMT1* 3’UTR (determined with starBase) and that a dual-luciferase reporter assay demonstrated that miR-21 did not directly target the 3’UTR of *DNMT1* (Fig. 5H), we speculated that miR-21 suppressed DNMT1 expression indirectly by inhibiting a transcription factor of DNMT1. To explore the underlying mechanism, the downregulated genes in GA-MSCs were filtered by overlapping them with predicted miR-21 targets (determined with starBase) and predicted DNMT1 transcription factors (determined with the PROMO database). Three candidates were identified as potential transcription factors of DNMT1 and potential targets of miR-21 (Fig. 5I), and SP1 has been reported to be a transcription factor of DNMT1 [49, 50]. We found that *SP1* knockdown reduced DNMT1 expression in human BM-MSCs (Fig. 5J) and that SP1 expression in BM-MSCs was downregulated after miR-21 overexpression (Fig. 5K), indicating that miR-21 might suppress DNMT1 expression by targeting SP1. To test whether miR-21 targets SP1 directly, 3’UTR seed sequence mutations and 3’UTR luciferase assays were conducted. *SP1* luciferase activity in miR-21-transfected BM-MSCs was decreased (Fig. 5L), indicating that *SP1* is a direct target of miR-21. In addition, SP1 overexpression reversed the inhibitory effect of miR-21 on DNMT1 (Fig. 5M), indicating that miR-21 indirectly regulated DNMT1 expression by targeting SP1 in human BM-MSCs. Moreover, we found that miR-21 also inhibited DNMT1 expression in mouse BM-MSCs (Fig. S5F), although the underlying mechanism was different from that in human BM-MSCs. Mouse miR-21 inhibited DNMT1 expression by targeting the 3’UTR of DNMT1 directly (Fig. S5G).

Overall, we found that upregulated miR-21 suppressed DNMT1 expression indirectly by inhibiting SP1 in human BM-MSCs, which resulted in a decreased DNA methylation level and increased miR-21 production, contributing to the formation of an miR-21/SP1/DNMT1 positive feedback loop. In addition, upregulated miR-21 decreased the DNA methylation level in mouse BM-MSCs by targeting DNMT1 directly, leading to further upregulation of miR-21.

### Modified exosomes loaded with miR-21 inhibitors improved PD-1 blockade therapy

**Fig. 6:**
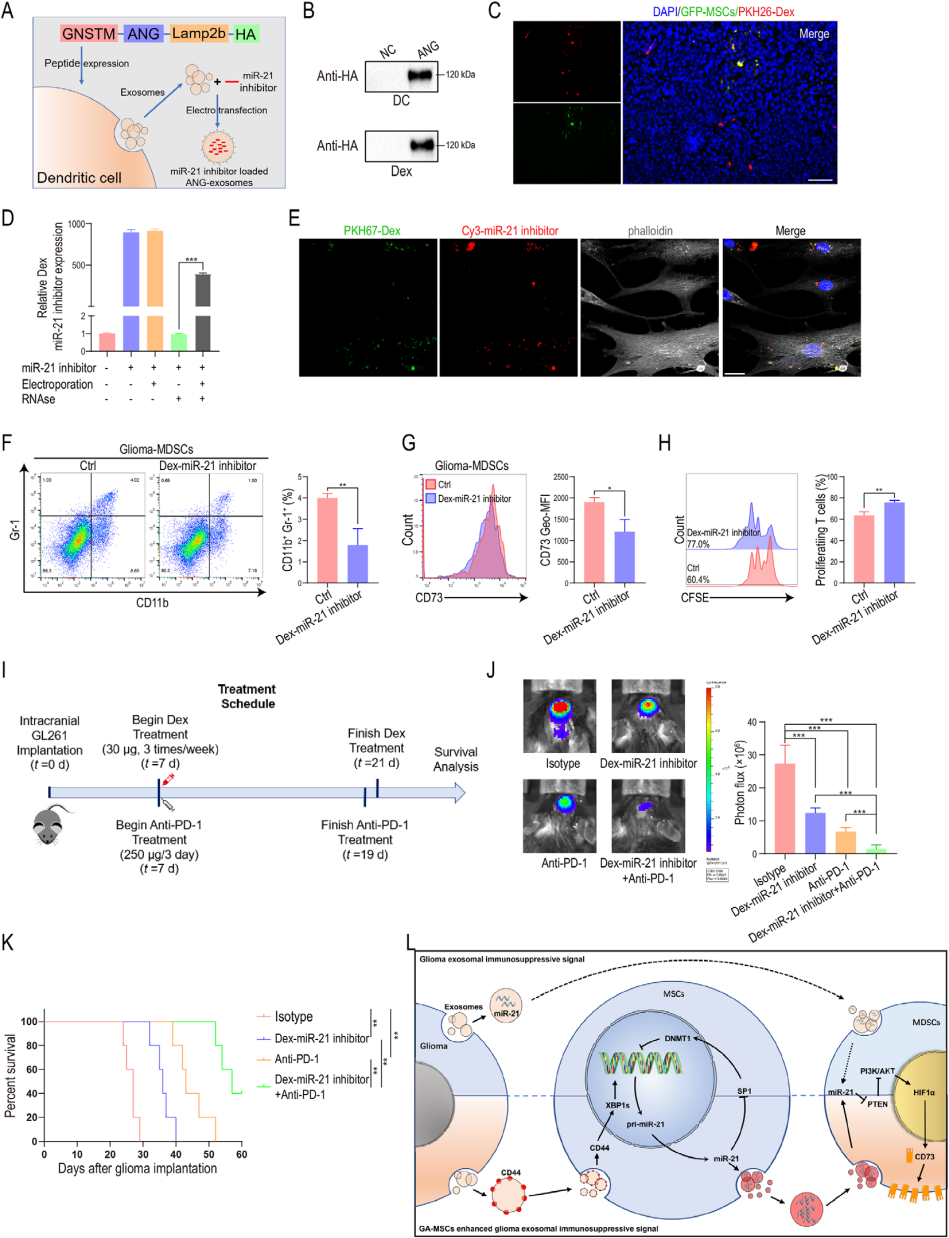
Modified exosomes loaded with miR-21 inhibitor improved PD-1 blockade therapy **(A)** Schematic diagram of DNA plasmid construction and the process used to produce modified exosomes loaded with miR-21 inhibitor. **(B)** DC were transfected with the GNSTM-ANG-Lamp2b-HA plasmid, and the expression of HA in DC and DC-derived exosomes (Dex) was measured by western blotting. **(C)** ANG-modified Dex was labeled with PKH26 (red) and injected intravenously into mice implanted with GL261 and GFP-labeled MSCs (green) in situ. The uptake of Dex by MSCs was observed using a fluorescence microscope. Scale bar = 50 μm. **(D)** Relative expression of miR-21 inhibitor in Dex without electroporation, with electroporation, without electroporation and with RNase treatment, or with electroporation and RNase A treatment. **(E)** Cy3-labeled miR-21 inhibitor (red) were loaded into PKH67-labeled ANG-modified Dex (green) by electroporation and used to treat GA-MSCs. Confocal microscopy showed the uptake of Dex by MSCs. Scale bar = 20 μm. **(F, G)** ANG-modified Dex containing miR-21 inhibitor were injected intravenously into GL261-bearing mice. The percentage of Gr-1^+^CD11b^+^ MDSCs and the expression of CD73 on MDSCs infiltrating in glioma tissues in mice were measured by flow cytometry. **(H)** Glioma-infiltrating MDSCs were cocultured with CFSE-labeled splenocytes from normal C57BL/6 mice, and CD8^+^ T-cell proliferation was measured using flow cytometry. **(I)** Schematic diagram of the schedule for glioma implantation and drug treatment. ANG-modified Dex (30 µg/mouse/time) containing miR-21 inhibitor were injected intravenously three times a week for 2 weeks. Anti-PD-1 antibodies were intraperitoneally injected into the mice (250 µg/mouse/time) on days 7, 10, 13, 16 and 19 after glioma implantation. **(J)** Tumor volume was evaluated using bioluminescence imaging, and the luminescence quantification is shown. **(K)** The survival curves of glioma-bearing mice are shown. Statistical significance was determined by the log-rank test. **(L)** Proposed working model of the miR-21/SP1/DNMT1 positive feedback loop in GA-MSCs induced by glioma exosomal CD44. The data are presented as the mean ± SD; *p < 0.05, **p < 0.01, ***p < 0.001

Having demonstrated the important role of miR-21 in mediating the formation of an immunosuppressive glioma microenvironment through the formation of a miR-21/SP1/DNMT1 positive feedback loop in MSCs and promotion of CD73 expression on MDSCs, we sought to determine whether targeting miR-21 could prevent glioma progression. In recent years, a modified exosome-based drug delivery system was designed for treating gliomas, and satisfactory results were obtained [51, 52]. Low-density lipoprotein receptor protein 1 (LRP1) is highly expressed on brain capillary endothelial and glioma cells, and angiopep-2 (ANG) is a type of peptide with a high affinity for LRP1. We and other researchers have reported that ANG peptide-modified engineered exosomes can cross the blood‒brain barrier and target the glioma microenvironment [36]. To produce glioma microenvironment-targeting and miR-21-inhibitor-carrying exosomes, we designed a modified exosome production process (Fig. 6A). The GNSTM-ANG-Lamp2b-HA plasmid was constructed and transfected into mouse dendritic cells (DC). A glycosylation sequence (GNSTM) was used to stabilize the ANG peptide. The targeting peptide ANG was fused to the N-terminus of the Lamp2b protein, an exosomal membrane protein. An HA-tag was added to the C-terminus of ANG-Lamp2b for detection by western blotting to verify successful transfection. The expression of HA-tag was detected in DC and DC-derived exosomes (Dex) (Fig. 6B). To validate whether modified Dex could be internalized by MSCs infiltrating in glioma, PKH26-labeled Dex were injected intravenously into C57BL/6 mice coimplanted in situ with GL261 cells and GFP-tagged MSCs. The internalization of exosomes by GA-MSCs was observed (Fig. 6C).

Next, miR-21 inhibitor was loaded into the modified exosomes by electroporation. We found that the exosome markers Flotillin-1, TSG101 and CD9 were detected in the Dex loaded with miR-21 inhibitor, and the negative marker Calnexin was absent (Fig. S6A). There was no difference in morphology or diameter between the Dex and Dex loaded with miR-21 inhibitor (Fig. S6B, C). The expression of the miR-21 inhibitor in Dex was significantly increased after electroporation (Fig. 6D). Treatment of electroporated Dex with RNase led to a degradation of about 60% miR-21 inhibitor, relative to untreated Dex, whereas, the same amount of miR-21 inhibitor in an unelectroporated mixture with Dex was completely degraded (Fig. 6D). This data suggests that approximately 40% of the miR-21 inhibitor were loaded into Dex by electroporation and protected from the RNase. Furthermore, Cy3-tagged miR-21 inhibitor were electroporated into PKH67-labeled modified Dex, and the internalization of the miR-21 inhibitor-Dex complex by GA-MSCs was observed using confocal microscopy (Fig. 6E). We also found that Dex loaded with miR-21-5p inhibitor increased DNMT1 expression in GA-MSCs (Fig. S6D), further validating the loading of miR-21 inhibitor in Dex. To evaluate the therapeutic effect, the miR-21 inhibitor-Dex complex was injected intravenously into C57BL/6 mice implanted in situ with GL261 cells, and we found that the percentage of MDSCs, CD73 expression on MDSCs and T-cell-suppressing function of MDSCs infiltrating in glioma were reduced (Fig. 6F-H).

Reportedly, CD73 is a combination therapy target that can improve the anti-GBM immune responses induced by anti-PD-1 immunotherapy [53], and our results demonstrated that the miR-21 inhibitor-Dex complex could inhibit CD73 expression on MDSCs in the glioma microenvironment. Therefore, we next examined whether the combination of the miR-21 inhibitor-Dex complex with anti-PD-1 mAb could confer superior antiglioma activity. miR-21 inhibitor-Dex and anti-PD-1 mAb were used to treat C57BL/6 mice implanted in situ with GL261 (Fig. 6I). We found that the combination of the anti-PD-1 mAb with the miR-21 inhibitor-Dex complex produced the strongest tumor inhibition effect of the treatments (Fig. 6J). In addition, compared to treatment with either the miR-21 inhibitor-Dex complex or anti-PD-1 mAb alone, combination therapy extended survival (Fig. 6K). Moreover, we found that miR-21 inhibitor-Dex complex treatment increased DNMT1 expression in CD45^−^Ter-119^−^ PDGFR-α^+^Sca-1^+^ mouse MSCs [54–56] infiltrating in glioma (Fig. S7A, B), indicating that the miR-21 inhibitor disrupted the miR-21/DNMT1 positive feedback loop in MSCs. We also found that CD73 expression on MDSCs infiltrating in glioma tissue was decreased after miR-21 inhibitor-Dex treatment (Fig. S7C, D). Furthermore, we found the percentage of CD8^+^IFN-γ^+^ cells was increased after miR-21 inhibitor-Dex treatment, and the combination of the anti-PD-1 mAb with the miR-21 inhibitor-Dex complex induced the most significant increase of CD8^+^IFN-γ^+^ cells (Fig. S7E), indicating that the miR-21 inhibitor-Dex complex enhanced the effects of PD-1 blockade immunotherapy. In conclusion, our results indicated that modified Dex loaded with miR-21-5p inhibitor improved PD-1 blockade therapy by disrupting the positive feedback loop in GA-MSCs.

## Discussion

The existence of an immunosuppressive microenvironment is a well-recognized feature of glioma and drives immunotherapy resistance [53]. The formation of an immunosuppressive microenvironment in glioma depends on communication among glioma cells, immune cells and stromal cells [57]. Cytokines, cell-surface receptors and exosomes are involved in this communication to transmit immunosuppressive signals [58]. In our previous studies, we reported that exosomal immunosuppressive signals from glioma could directly regulate the functions of immune cells, including MDSCs and tumor-associated macrophages, to promote immunosuppression [12, 28, 29]. Here, we found that glioma-derived exosomal immunosuppressive signals promoted the formation of an immunosuppressive microenvironment by modifying the function of MSCs and regulating the function of MDSCs indirectly.

MSCs are important components of the tumor microenvironment and contribute to the formation of an immunosuppressive microenvironment by regulating the functions of multiple immune cells [59]. The percentage of MSCs infiltrating glioma tissues is negatively correlated with glioma prognosis [11]. Reportedly, GA-MSCs promote the growth of glioma stem cells through exosomal miR-1587 [60], but there is no research on the immunoregulatory effect of GA-MSCs in glioma. In this study, we found that GA-MSCs upregulated CD73 expression on MDSCs via exosomal miR-21 through the PTEN/PI3K/AKT/HIF-1α pathway, promoting the formation of an immunosuppressive microenvironment and the progression of glioma.

Considering their strong tropism toward glioma, low immunogenicity and easy accessibility, MSCs have been developed as vehicles for the targeted delivery of drugs to glioma in recent years [61]. Reportedly, MSCs loaded with paclitaxel [62], miRNA [63, 64], IL12/IL7 [65] or oncolytic virus [66] can prolong the survival of glioma-bearing mice. However, some studies have indicated that MSCs educated by glioma promote glioma cell proliferation [67, 68]. Our study also proved that glioma-associated MSCs promoted glioma progression by promoting the formation of an immunosuppressive microenvironment, revealing the potential risk related to MSC-based therapies for GBM. To reduce the risk, MSC-derived exosomes [69] and nanoparticles coated with MSC membrane [70] have been generated, which maintain the glioma homing ability and avoid being reprogrammed by glioma, thus being developed as potential strategies for glioma-targeting drug delivery.

Previously, we reported that glioma-derived exosomal miR-21 promoted the immunosuppressive function of MDSCs [35]. In this study, we found that MSC-derived exosomal miR-21 also promoted the immunosuppressive function of MDSCs and that the miR-21 level in GA-MSCs-derived exosomes was more than six times higher than that in glioma-derived exosomes (Fig. S3J). Further studies were performed, and we found that the enrichment of miR-21 in GA-MSCs exosomes was caused by the miR-21/SP1/DNMT1 positive feedback loop triggered by glioma exosomal CD44. This finding indicated that GA-MSCs were an important part of the immunosuppressive MDSCs pathway induced by glioma exosomes, which could amplify the exosomal immunosuppressive signaling from glioma through a positive feedback loop (Fig. 6L). The treatment strategy of targeting GA-MSCs has great potential to relieve the immunosuppression in glioma.

Reportedly, miR-21 can bind directly to DNMT1 and inhibit its enzymatic activity [71]. The upregulation of miR-21 in MSCs could inhibit the enzymatic activity of DNMT1 on the one hand and downregulate the expression of DNMT1 by inhibiting SP1 on the other hand, which together led to a decreased level of DNA methylation that further promoted the elevated expression of miR-21.

miR-21 plays important roles in promoting tumor progression. Reportedly, miR-21 can promote the proliferation of glioma cells, enhance resistance to radiotherapy and chemotherapy, promote M2 polarization of macrophages, induce the differentiation and activation of MDSCs, and inhibit T-cell activation [35, 72–74]. Targeting miR-21 can inhibit tumor progression. Considering the presence of the blood‒brain barrier, many drugs have a limited ability to treat glioma, while exosome-loaded drugs have been reported to be effective in the treatment of glioma [75]. Exosomes exhibit high stability in the peripheral blood and have a hydrophilic core that can transport soluble drugs [76]. The low immunogenicity of exosomes also makes it difficult for them to be cleared by the immune system [77]. To achieve drug delivery to the brain and break the positive feedback loop, Dex were modified with ANG and loaded with miR-21 inhibitor. We found that the modified Dex containing the miR-21 inhibitor could be internalized by GA-MSCs and prevented the formation of an immunosuppressive microenvironment in glioma.

In recent years, Dex have attracted more attention due to their immunoregulatory ability in cancer treatment. Dex were found to possess MHC-I and MHC-II antigen-presenting molecules and CD86 costimulatory molecules, which could potentially stimulate T cells and inhibit tumor growth [78]. Dex-based phase I and II clinical trials have been conducted in several cancers, showing the feasibility and safety of the approach [79–81]. However, the existence of an immunosuppressive tumor microenvironment limits the effect of Dex [82]. We found that Dex loaded with miR-21 inhibitor prevented the formation of an immunosuppressive microenvironment by suppressing the function of MDSCs, providing an option for improving the immunotherapeutic effects of Dex. In addition, it has been reported that Dex derived from tumor peptide-stimulated DC and tumor-derived exosome-stimulated DC are more powerful in stimulating the antitumor immune response [83], suggesting that Dex derived from DC stimulated with glioma-derived exosomes could be a better choice to carry miR-21 inhibitor and may exert stronger antiglioma effects, though this needs to be further investigated.

Our results indicated that Dex loaded with a miR-21 inhibitor prolonged the survival of glioma-bearing mice by disrupting the positive feedback loop in MSCs (Fig. S7B); however, the improvement in therapeutic effect was not very significant (Fig. 6K), although the infiltration of MDSCs in glioma and CD73 expression on MDSCs were significantly decreased after miR-21 inhibitor-Dex treatment (Fig. S7C-D). Reportedly, PD-L1 is overexpressed in glioma cells [84] and MDSCs [29] in the glioma microenvironment, binding to PD-1 on the surface of activated T cells and leading to an immunosuppressive effect, which might have limited the immunotherapy efficacy of the miR-21 inhibitor-Dex complex. Therefore, combination of the miR-21 inhibitor Dex with anti-PD-1 therapy significantly improved the immunotherapy efficacy and increased the percentage of CD8^+^IFN-γ^+^ cells (Fig. S7E), leading to significantly prolonged survival in mouse model (Fig. 6K).

Immune checkpoint therapy with anti-PD-1 has revolutionized the treatment of many solid tumors. However, in a phase III trial, anti-PD-1 therapy failed to increase the survival of patients with GBM [85, 86]. The existence of an immunosuppressive microenvironment limits the efficacy of anti-PD-1 therapy. To improve antitumor immune responses to anti-PD-1 in GBM, many clinical studies have explored combining anti-PD-1 with other immune checkpoint blockade agents, such as IDO1 inhibitors (ClinicalTrials.gov identifier: NCT03707457) and anti-lymphocyte-activation gene 3 (LAG-3) monoclonal antibodies (NCT02658981). Reportedly, CD73 is a specific molecule that can be targeted to improve the effect of anti-PD-1 therapy in glioma [25]. In addition, targeting MDSCs also improves the efficacy of PD-1 blockade in GBM [87]. In our study, we found that modified Dex containing miR-21 inhibitor could disrupt the miR-21/SP1/DNMT1 positive feedback loop in GA-MSCs, leading to a decrease in MDSCs infiltration and CD73 expression on MDSCs, and synergizing with an anti-PD-1 monoclonal antibody (mAb) to prolong the survival of glioma-bearing mice. Considering that phase I and phase II clinical trials of Dex have been performed in many cancer types [79, 81], the combination of Dex with a clinically available treatment (anti-PD-1 therapy) would allow for accelerated translation of these preclinical results into early-phase human clinical trials.

## Conclusion

In summary, we elucidated that GA-MSCs play a key role in promoting the formation of an immunosuppressive glioma microenvironment by amplifying glioma exosomal immunosuppressive signaling through the miR-21/SP1/DNMT1 positive feedback loop. Targeting miR-21 in GA-MSCs with modified Dex prevented the formation of an immunosuppressive microenvironment and improved the efficacy of PD-1 blockade therapy, therefore providing a promising strategy for improving immunotherapy for GBM patients.

## Electronic supplementary material

Below is the link to the electronic supplementary material.

Supplementary Material 1

## Data Availability

The array data support the findings of this study have been deposited in the NCBI Sequence Read Archive database under accession code PRJNA814416, PRJNA814429 and PRJNA816564. The remaining data are available within the Article, Supplementary Information and available from the authors upon request.

## Abbreviations

MSCs: Mesenchymal stem cells
GA-MSCs: glioma-associated mesenchymal stem cells
MDSCs: myeloid-derived suppressor cells
DC: dendritic cells
GDEs: glioma-derived exosomes
Dex: Dendritic cell-derived exosomes
GBM: Glioblastoma multiforme
DNMT1: DNA methyltransferase 1
MSP: Methylation-specific PCR
ChIP: Chromatin immunoprecipitation
ER: endoplasmic reticulum
ANG: Angiopep-2
CM: Culture medium
EXO: exosomes
