# Supplementary material for: Mesenchymal stem cells, as glioma exosomal immunosuppressive signal multipliers, enhance MDSCs immunosuppressive activity through the miR-21/SP1/DNMT1 positive feedback loop
Source: J Nanobiotechnology. 2023 Jul 22;21:233. doi: 10.1186/s12951-023-01997-x (PMC10362641; doi:10.1186/s12951-023-01997-x)
Supplement: Supplementary file 1 — Supplementary Material 1 [file 12951_2023_1997_MOESM1_ESM.pdf]

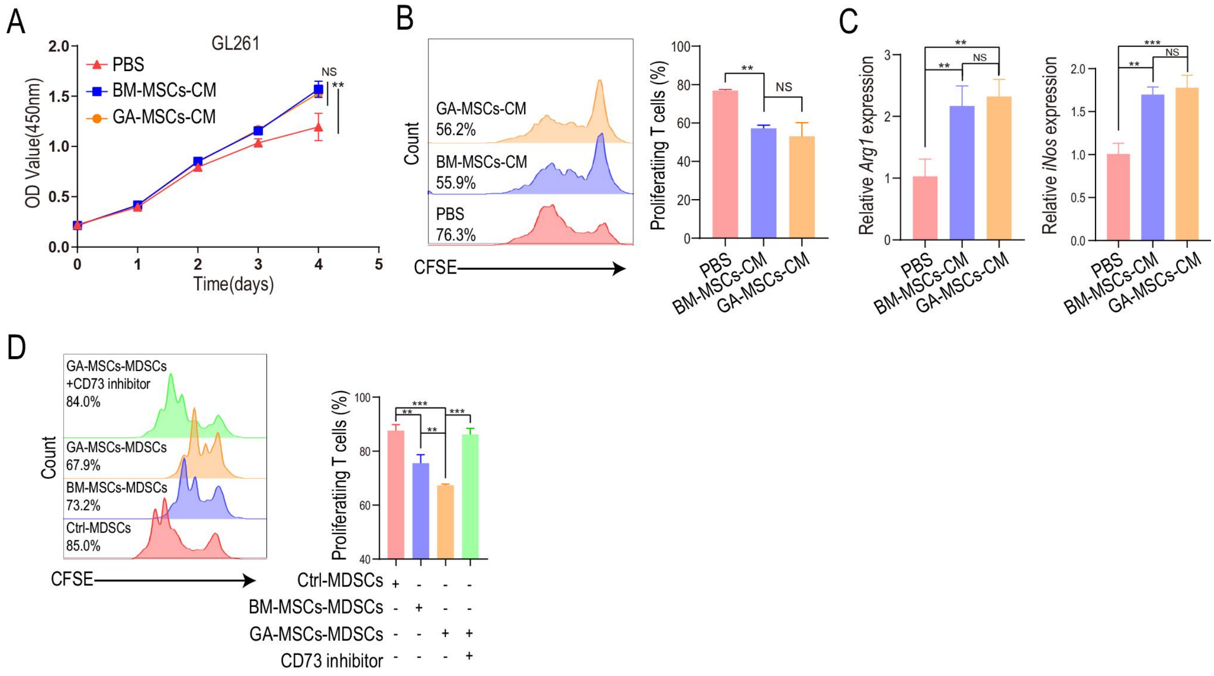

Figure S1 GA-MSCs promoted glioma growth by inducing CD73 expression on MDSCs

(A) PBS, mouse BM-MSCs- or GA-MSCs-derived culture medium (CM) was used to stimulate GL261. The proliferation of GL261 was evaluated using CCK8 assays. **(B)** PBS, mouse BM-MSCs- or GA-MSCs-derived CM was used to treat CFSE-labeled splenocytes isolated from normal C57BL/6 mouse. The proliferation of $CD8^+$ lymphocytes was evaluated by measuring the CFSE dilution signal. **(C)** PBS, mouse BM-MSCs- or GA-MSCs-derived CM was used to treat mouse bone marrow cells, and the expression of *Arg1* and *iNos* in MDSCs were measured using qRT-PCR. **(D)** Mice were implanted with GL261 (Ctrl group), GL261 mixed with bone marrow MSCs (BM-MSCs groups) or GL261 mixed with glioma-associated MSCs (GA-MSCs group). The glioma-infiltrating $Gr\text{-}1^+CD11b^+$ MDSCs were isolated and co-cultured with CFSE-labeled splenocytes from normal C57BL/6 mouse in the absence or presence of CD73 inhibitor. After 72 h of coculture, $CD8^+$ T cell proliferation was evaluated by measuring the CFSE dilution signal. The data are presented as the mean ± SD; $*p < 0.05$, $**p < 0.01$, and $***p < 0.001$.

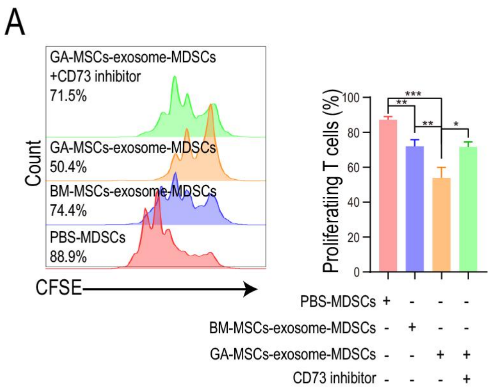

Figure S2 GA-MSCs-derived exosomes promote T cell suppressive ability of MDSCs *in vivo*

(A) PBS, mouse BM-MSCs-derived exosomes or GA-MSCs-derived exosomes was intravenously injected into C57BL/6 mice. The splenic Gr-1^+CD11b^+ MDSCs were isolated and co-cultured with CFSE-labeled splenocytes from normal C57BL/6 mice in the absence or presence of CD73 inhibitor. After 72 h of co-culture, $CD8^+$ T cell proliferation was evaluated by measuring the CFSE dilution signal. The data are presented as the mean ± SD; *p <0.05, **p < 0.01, and ***p < 0.001.

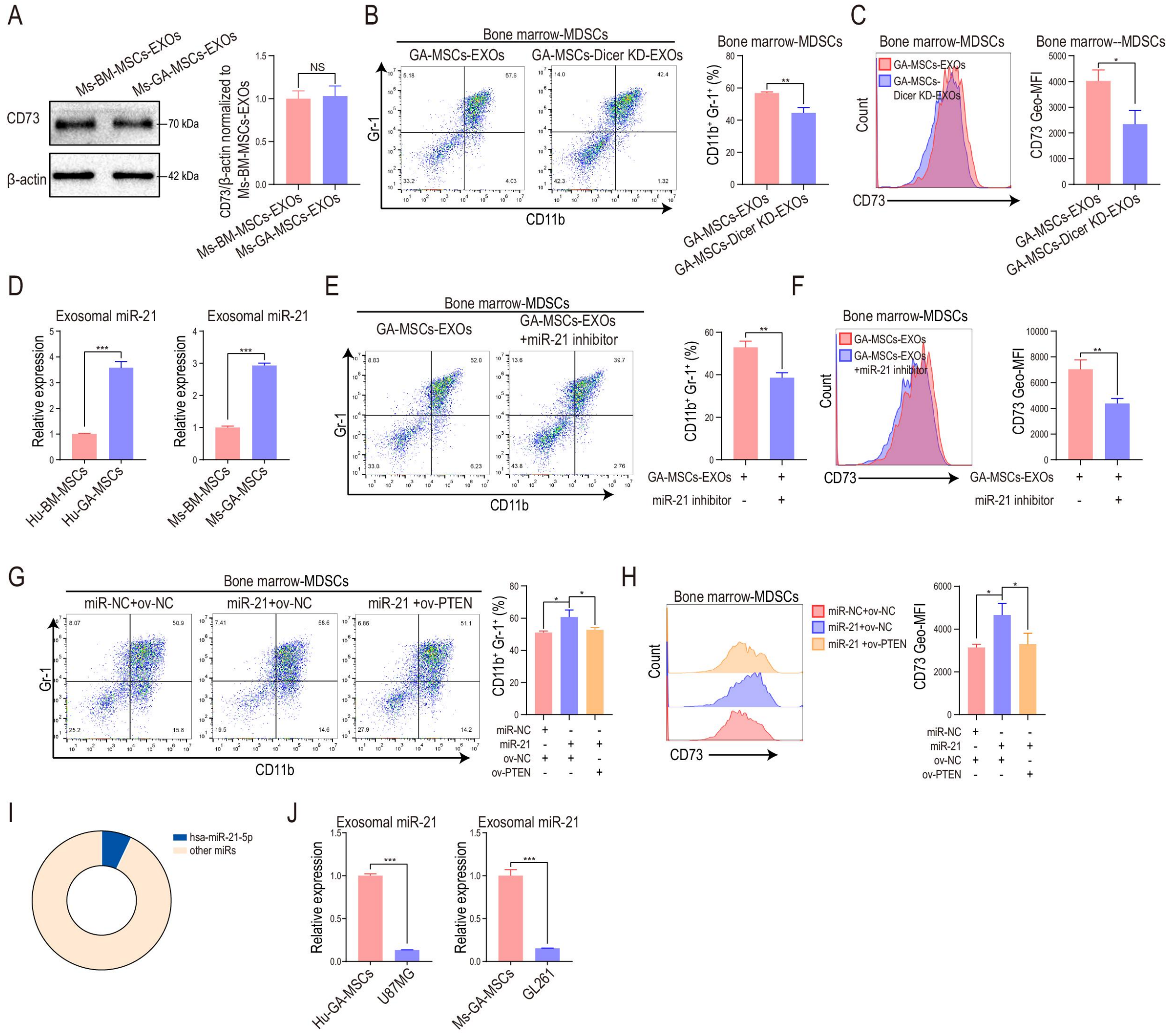

Figure S3 Mouse GA-MSCs-derived exosomal miR-21 upregulated CD73 expression on MDSCs

(A) The CD73 expression in exosomes derived from mouse BM-MSCs and GA-MSCs was measured using western blot. Quantification of the fold change in the CD73/β-actin ratio (normalized to BM-MSCs-EXOs) is shown. **(B, C)** Exosomes derived from mouse GA-MSCs with or without *DICER* knockdown were used to stimulate mouse bone marrow cells. The percentage of Gr-1$^+$CD11b$^+$ MDSCs and the expression of CD73 on MDSCs were measured by flow cytometry. **(D)** The expression of miR-21 was measured in human (Hu) and mouse (Ms) BM-MSCs and GA-MSCs-derived exosomes using qRT–PCR assays. **(E, F)**

Mouse bone marrow cells were stimulated with mouse GA-MSC-derived exosomes and transfected with miR-21 inhibitor. The percentage of Gr-1$^+$CD11b$^+$ MDSCs and the expression of CD73 on MDSCs were measured by flow cytometry. **(G, H)** The percentage of MDSCs and CD73 expression on MDSCs induced by miR-NC or miR-21 mimics and nonsense sequence or PTEN overexpression plasmid were measured using flow cytometry. **(I)** The percentage of miR-21 expression in total miRNAs expression in U87MG-derived exosomes. **(J)** The expression of miR-21 in exosomes derived from U87MG and human GA-MSCs or from GL261 and mouse GA-MSCs was measured using qRT-PCR. The nematode miRNA cel-miR-39 was used as an external reference. The data are presented as the mean ± SD; *$p < 0.05$, **$p < 0.01$, and ***$p < 0.001$.

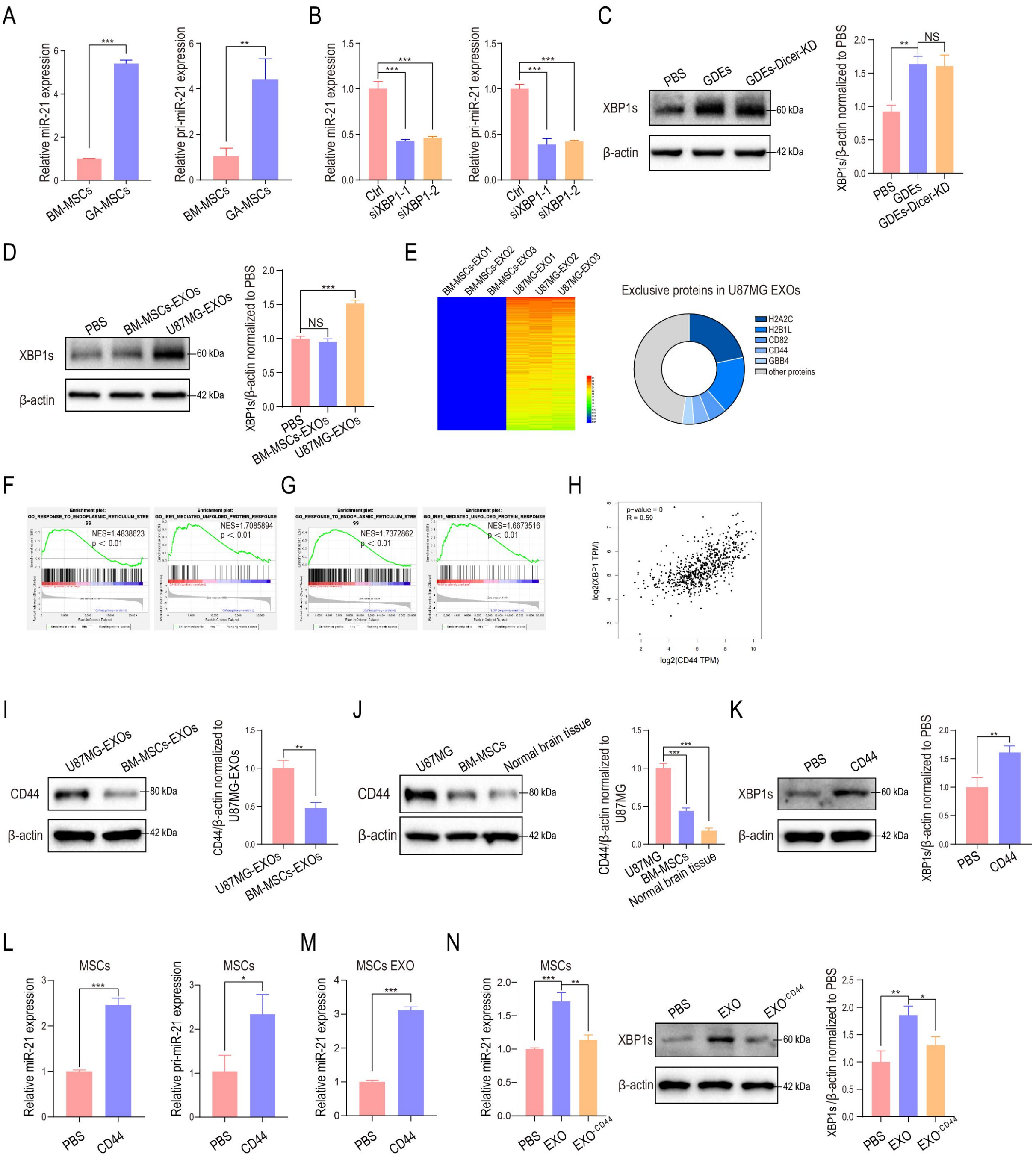

Figure S4 Glioma-derived exosomal CD44 promote XBP1s expression in BM-MSCs

(A) The expression of miR-21 and pri-miR-21 was measured in human BM-MSCs and GA-MSCs using qRT-PCR. **(B)** The expression of miR-21 and pri-miR-21 was measured in control and *XBP1* knockdown human BM-MSCs. **(C)** Exosomes derived from U87MG with or without *DICER* knockdown were used to treat BM-MSCs. The XBP1s expression was measured using western blot. Quantification of the fold change in the XBP1s/β-actin ratio

(normalized to PBS-treated cells) is shown. **(D)** Exosomes derived from human BM-MSCs or U87MG were used to treat BM-MSCs. The XBP1s expression was measured using western blot. Quantification of the fold change in the XBP1s/β-actin ratio (normalized to PBS-treated cells) is shown. **(E)** The exosomal protein profile data of U87MG and human BM-MSCs were analyzed and the proteins highly expressed in U87MG-derived exosomes but hardly expressed in BM-MSCs-derived exosomes were shown. **(F)** GSEA analysis of transcriptome sequencing data indicates GA-MSCs are positively correlated with endoplasmic reticulum stress and UPR activation compared to BM-MSCs. **(G)** GSEA analysis indicates the expression of CD44 is positively correlated with endoplasmic reticulum stress and UPR activation. **(H)** The correlation between XBP1 and CD44 in TCGA glioma database. **(I)** The expression of CD44 in human BM-MSCs- and U87MG-derived exosomes were measured using western blot. Quantification of the fold change in the CD44/β-actin ratio (normalized to U87MG-derived exosomes) is shown. **(J)** The expression of CD44 in human glioma cell line U87MG, human BM-MSCs and normal human brain tissues (taken from trauma patients who underwent partial resection of the normal brain as decompression treatment for severe head injuries) were measured using western blot. Quantification of the fold change in the CD44/β-actin ratio (normalized to U87MG cells) is shown. **(K)** The expression of XBP1s was measured in BM-MSCs treated with recombinant CD44 protein. Quantification of the fold change in the XBP1s/β-actin ratio (normalized to PBS-treated cells) is shown. **(L)** The expression of miR-21 and pri-miR-21 was measured in human BM-MSCs treated with recombinant CD44 protein. **(M)** The recombinant CD44 protein was used to treat human BM-MSCs and the expression of exosomal miR-21 was measured. **(N)** Exosomes were

isolated from culture medium of U87MG with or without *CD44* knockdown and used to stimulate BM-MSCs. The protein level of XBP1s and the cellular miR-21 expression in BM-MSCs were measured. The data are presented as the mean ± SD; *p <0.05, **p < 0.01, and ***p < 0.001.

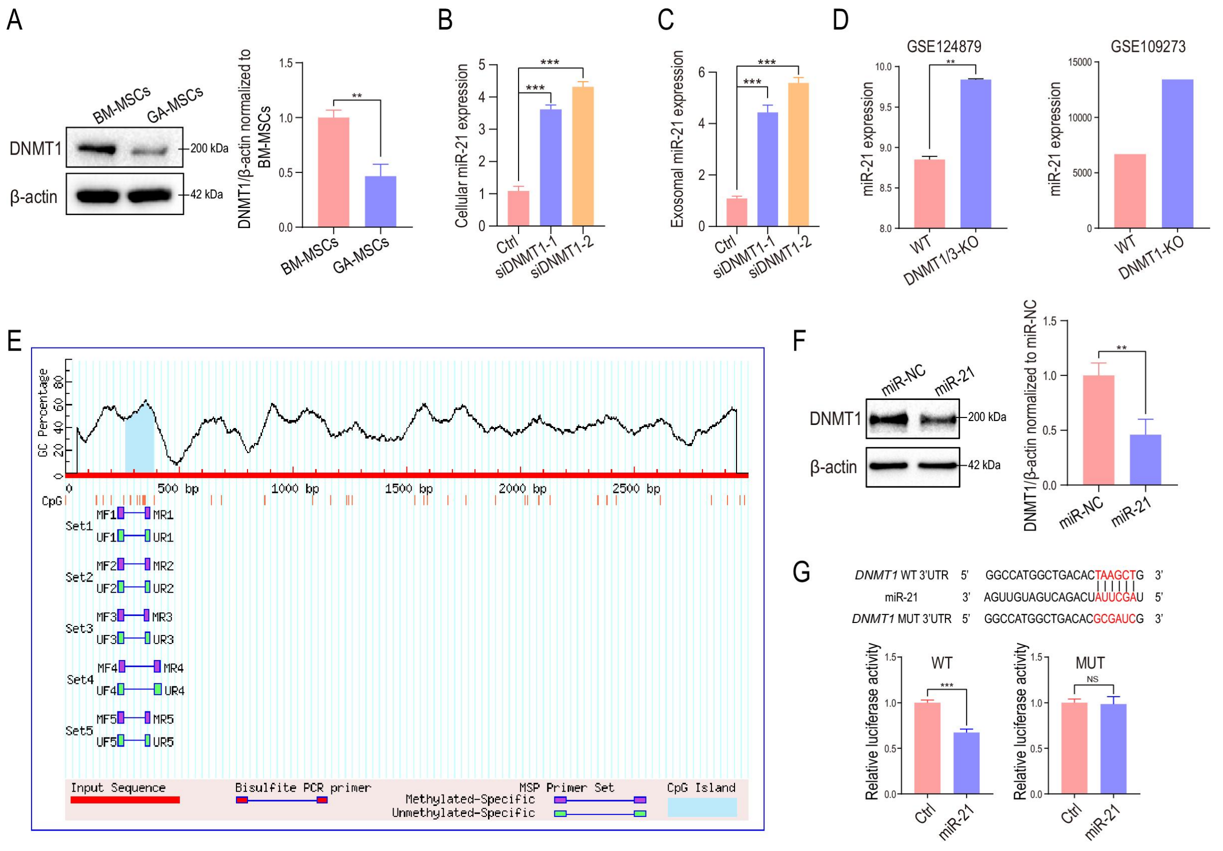

Figure S5 The miR-21/DNMT1 positive feedback loop in mouse MSCs promoted miR-21 expression

(A) The DNMT1 expression in mouse BM-MSCs and GA-MSCs was measured using western blot. Quantification of the fold change in the DNMT1/β-actin ratio (normalized to BM-MSCs) is shown. **(B)** The miR-21 expression was measured in control and *DNMT1* knockdown mouse BM-MSCs. **(C)** The expression of miR-21 was measured in control and *DNMT1* knockdown mouse BM-MSCs derived exosomes. **(D)** The miR-21 expression was analyzed in *DNMT1/3* knockdown mouse cells (GSE124879) and *DNMT1* knockdown mouse cells (GSE109273). **(E)** The CpG island in the promoter region of miR-21 was predicted using MethPrimer. **(F)** The DNMT1 expression in mouse BM-MSCs transfected with miR-21 mimics was measured using western blot. Quantification of the fold change in the DNMT1/β-actin ratio (normalized to NC group) is shown. **(G)** BM-MSCs were

co-transfected with miR-21 and luciferase reporter containing 3'UTR region of mouse *DNMT1*. The luciferase activity was measured. The data are presented as the mean ± SD; *p < 0.05, **p < 0.01, ***p < 0.001.

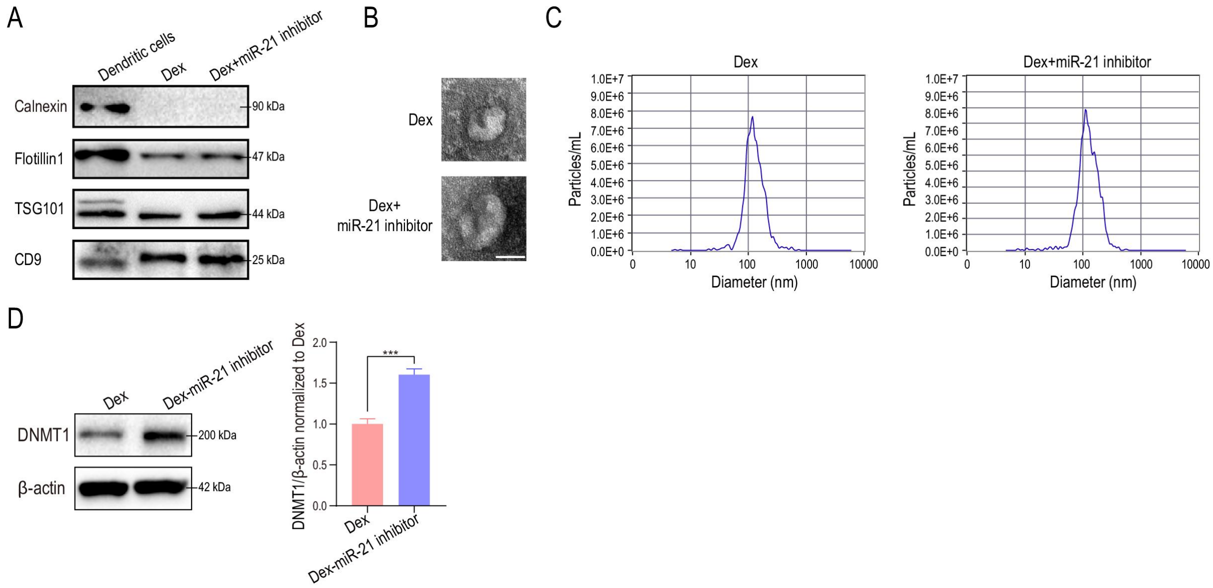

Figure S6. Characterization of Dex.

(A) Western blot analysis showing the expression of exosome markers Flotillin-1, TSG101 and CD9, and the absence of negative marker Calnexin in Dex and Dex loaded with miR-21 inhibitor. **(B)** Representative transmission electron microscopy images of Dex and Dex loaded with miR-21 inhibitors. Scale bar: 50 nm. **(C)** The diameter of Dex and Dex loaded with miR-21 inhibitors was analyzed by ZetaView system. **(D)** The DNMT1 expression in GA-MSCs treated with Dex or miR-21 inhibitor-Dex was measured using western blot. Quantification of the fold change in the DNMT1/β-actin ratio (normalized to Dex-treated cells) is shown. The data are presented as the mean ± SD; *p < 0.05, **p < 0.01, ***p < 0.001.

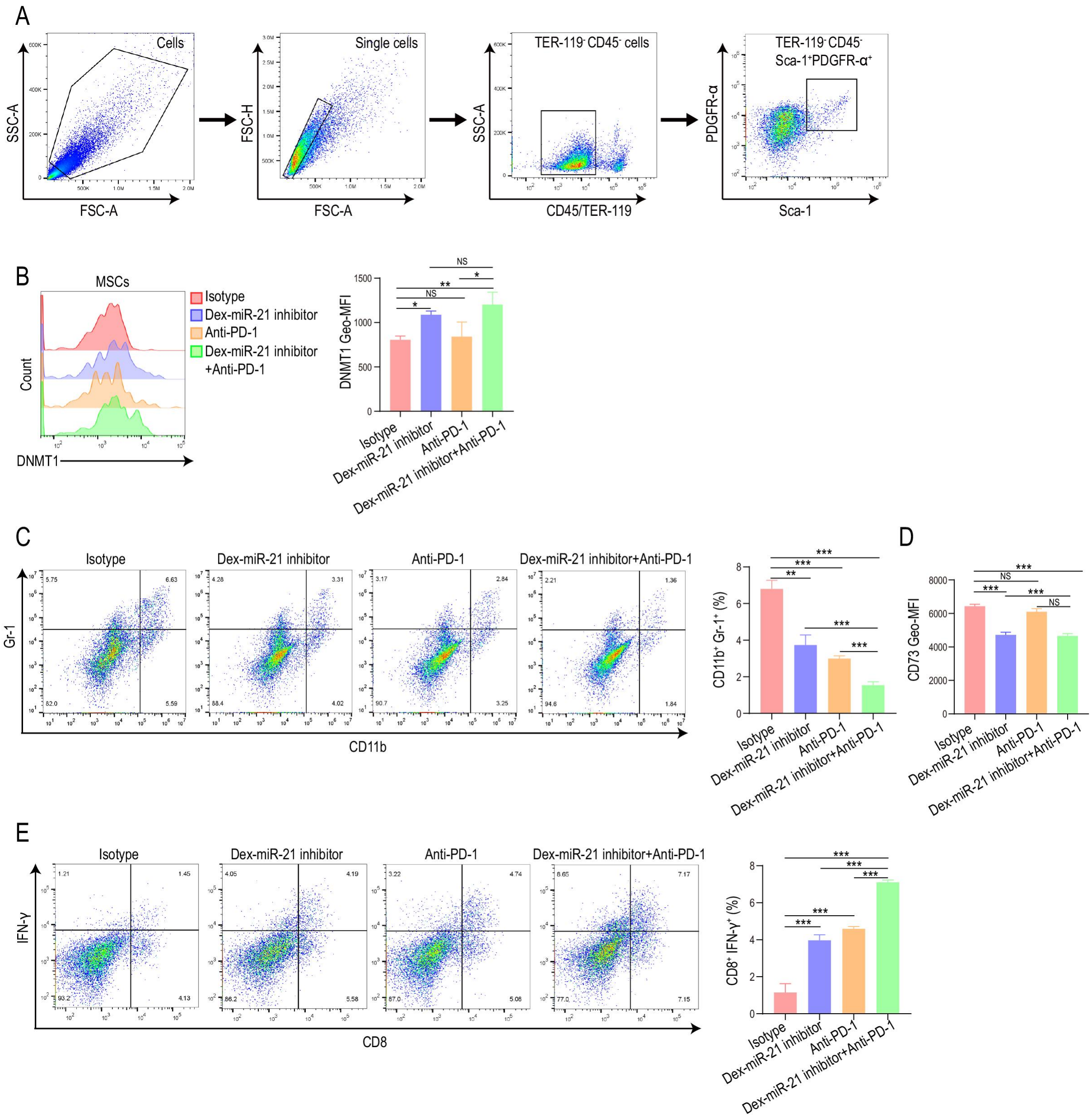

Figure S7. miR-21 inhibitor-Dex disrupted the miR-21/DNMT1 positive feedback loop in MSCs and improved PD-1 blockade therapy

(A) Gating strategy for identifying $CD45^{-}TER\text{-}119^{-}PDGFR\text{-}\alpha^{+}Sca\text{-}1^{+}$ MSCs cells infiltrating in glioma tissues. **(B)** miR-21 inhibitor-Dex and anti-PD-1 mAb were used to treat C57BL/6 mice implanted in situ with GL261. The expression of DNMT1 in MSCs infiltrating in glioma tissues in mice were measured by flow cytometry. **(C, D)** The percentage of $Gr\text{-}1^{+}CD11b^{+}$ MDSCs and the expression of CD73 on MDSCs infiltrating in glioma tissues in

mice were measured by flow cytometry. **(E)** The percentage of $CD8^{+}IFN\text{-}\gamma^{+}$ cells in glioma tissues in mice were measured by flow cytometry. The data are presented as the mean ± SD; $*p < 0.05$, $**p < 0.01$, $***p < 0.001$.

Table S1. siRNA, miRNA mimics and inhibitor used

si-NC	UUCUCCGAACGUGUCACGUTT
si*TP53*	GACUCCAGUGGUAAUCUACTT
si*EGR1*	ATAAGCCAAACAGUCACUUTT
si*DICER1*	GCCAAGGAAAUCAGCUAAATT
si*DNMT1*-1	CCAUGAGCACCGUUCUCCTT
si*DNMT1*-2	GGAGAACGGUGCUCAUGGTT
si*XBP1*-1	CACCCUGAAUUCAUUGUCU
si*XBP1*-2	CCAGGAGUUAAGACAGCGC
si*SP1*	CCAGCAACAUGGGAAUUAUTT

miRNA mimics NC	UUCUCCGAACGUGUCACGUTT
miR-26a-5p mimics	UUCAAGUAAUCCAGGAUAGGCU
miR-21-5p mimics	UAGCUUAUCAGACUGAUGUUGA
miR-10a-5p mimics	UACCCUGUAGAUCCGAAUUUGUG
let-7i-5p mimics	UGAGGUAGUAGUUUGUGCUGUU
let-7a-5p mimics	UGAGGUAGUAGGUUGUAUAGUU
miR-100-5p mimics	AACCCGUAGAUCCGAACUUGUG
miRNA inhibitor NC	CAGUACUUUUGUGUAGUACAA
mir-21-5p inhibitor	UCAACAUCAGUCUGAUAAGCUA

Table S2. Primers used

**Name**	**Forward primer**	**Reverse primer**
NT5E	GAAGTGAGGGGTGTGGACG	CCTTCCGCCCATCATCAGAA
ACTB	CATGTACGTTGCTATCCAGGC	CTCCTAATGTCACGCACGT
pri-miR-21	TTTTGTTTTGCTTGGGAGGA	AGCAGACAGTCAGGCAGGAT
MSP-M	GGGGATTTTGTGTATGTGTTATTAC	ATCCCAACACTTTAAAAAACCG
MSP-U	TGGGGATTTTGTGTATGTGTTATTAT	ATCCCAACACTTTAAAAAACCAA
XBP-1 CHIP	GGATGACGCACAGATTGTCCTA	TCAGAAGTCCCACATTTATCACCA
Actb	GGCTGTATTCCCCTCCATCG	CCAGTTGGTAACAATGCCATGT
Arg1	TGTCCCTAATGACAGCTCCTT	GCATCCACCCAAATGACACAT
Inos	CCTGCTTTGTGCGAAGTGTC	CCCAAACACCAAGCTCATGC
MiR-21 inhibitor	AGATGTGCGTCAACATCAGTCTG	TATGGTTGTTGACGACTGGTTGAC

Table S3. Antibodies used

Name	Company	Cat.
β-Actin	Cell Signaling Technology	8457
XBP1-s	Proteintech	24868-1-AP
DNMT1	Cell Signaling Technology	5032T
SP1	Santa Cruz Biotechnology	sc-420
PTEN	Proteintech	22034-1-AP
p-AKT	Cell Signaling Technology	4060
AKT	Cell Signaling Technology	4691
CD73	Proteintech	12231-1-AP
HIF-1α	Cell Signaling Technology	36169S
TSG101	Abcam	ab125011
CD9	System Biosciences	ExoAB-CD9A-1
Calnexin	Cell Signaling Technology	2679
Flotillin1	Proteintech	15571-1
